# Supplementary material for: Mutations in INPP5K, Encoding a Phosphoinositide 5-Phosphatase, Cause Congenital Muscular Dystrophy with Cataracts and Mild Cognitive Impairment
Source: Am J Hum Genet. 2017 Feb 9;100(3):523–36. doi: 10.1016/j.ajhg.2017.01.024 (PMC5339217; doi:10.1016/j.ajhg.2017.01.024)
Supplement: Document S1. Figures S1–S11 and Table S1 [file mmc1.pdf]

The American Journal of Human Genetics, Volume *100*

Supplemental Data

Mutations in *INPP5K*, Encoding a Phosphoinositide 5-Phosphatase, Cause Congenital Muscular Dystrophy with Cataracts and Mild Cognitive Impairment

Manuela Wiessner, Andreas Roos, Christopher J. Munn, Ranjith Viswanathan, Tamieka Whyte, Dan Cox, Benedikt Schoser, Caroline Sewry, Helen Roper, Rahul Phadke, Chiara Marini Bettolo, Rita Barresi, Richard Charlton, Carsten G. Bönnemann, Osório Abath Neto, Umbertina C. Reed, Edmar Zanoteli, Cristiane Araújo Martins Moreno, Birgit Ertl-Wagner, Rolf Stucka, Christian De Goede, Tamiris Borges da Silva, Denisa Hathazi, Margherita Dell'Aica, René P. Zahedi, Simone Thiele, Juliane Müller, Helen Kingston, Susanna Müller, Elizabeth Curtis, Maggie C. Walter, Tim M. Strom, Volker Straub, Kate Bushby, Francesco Muntoni, Laura E. Swan, Hanns Lochmüller, and Jan Senderek

Figure S1. Genome-wide linkage analysis of family A.

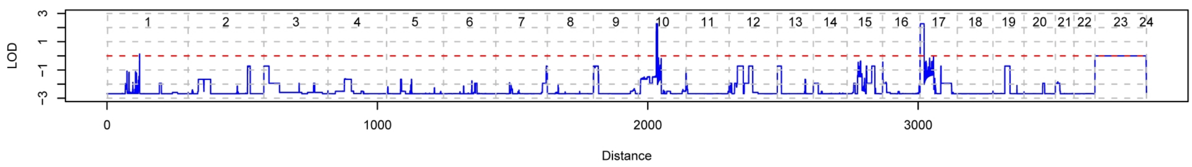

Genome-wide SNP genotyping was performed using the Illumina 300K chip (Illumina). Multipoint linkage was calculated with ALLEGRO [1] assuming autosomal recessive inheritance, a frequency of the deleterious allele of 0.001, and complete penetrance. The region of interest on chr10 spanned 7.18 Mb and contained 42 RefSeq genes. The candidate region on chr17 spanned 5.58 Mb and contained 98 RefSeq genes.

Figure S2. ***INPP5K*** **mutations identified in individuals with CMD and cataracts.**

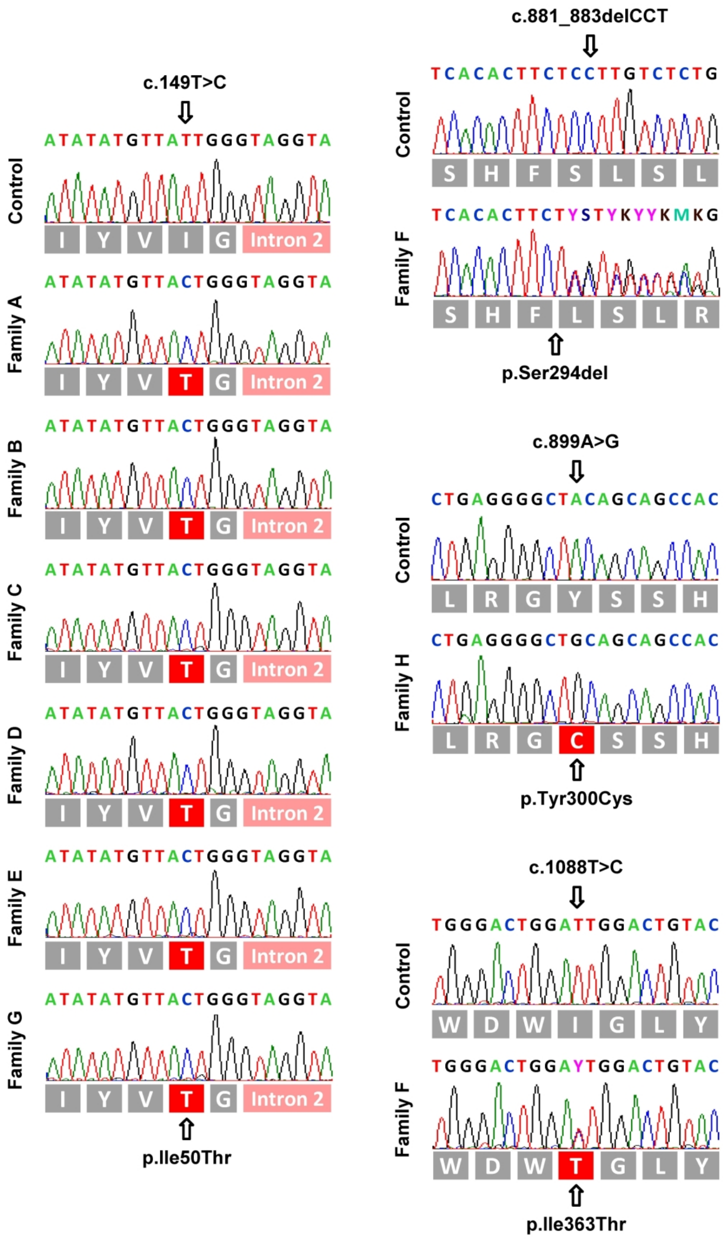

Sequencing electropherograms for comparison of nucleotide sequences of individuals affected with CMD and cataracts and unaffected control individuals.

Figure S3. Tissue distribution and localization of INPP5K.

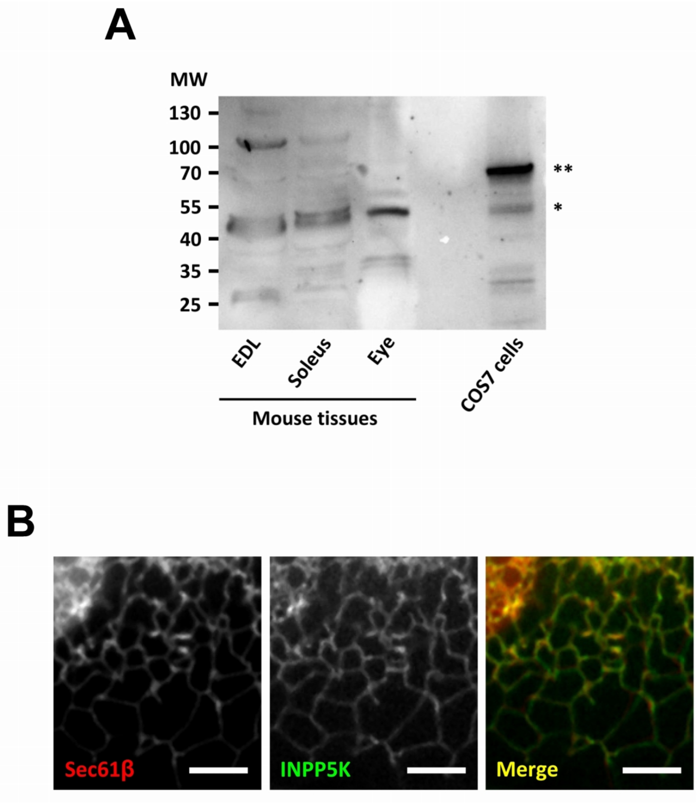

(A) Tissues from young adult C57BL/6J mice (P56) were homogenized with a rotor-stator homogenizer (TissueRuptor, Qiagen) in 1x PBS containing 1% SDS as well as protease (Complete Protease Inhibitor; Roche Diagnostics) and phosphatase inhibitors (PhosSTOP; Roche Diagnostics). Protein preparations were incubated at 95°C for 5 min, debris was removed by 5 min centrifugation at 14,000 rpm at 4°C, and protein concentrations in the supernatants were measured with a BCA protein assay (Thermo Fisher Scientific). Protein samples were separated by sodium dodecyl sulfate (SDS)-polyacrylamide gel electrophoresis and transferred to a nitrocellulose membrane (Protran; GE Healthcare). Transferred proteins were blocked for 1 hr at RT in TBS-T with 5% non-fat milk and then incubated overnight at 4°C with goat anti-INPP5K antibody (C19; Santa Cruz Biotechnology; 1:200). Subsequently, the blot was incubated with horseradish peroxidise (HRP)-conjugated donkey anti-goat IgG antibody (Jackson ImmunoResearch; 1:10,000) for 1 hr at RT. Bands were detected with an ECL Detection Kit (GE Healthcare). A lysate from COS-7 cells transfected with an expression vector coding for a GFP-INPP5K fusion protein was used as a control. EDL = extensor digitorum longus muscle; soleus = soleus muscle; eye = whole eye tissue; * = endogenous INPP5K; ** = GFP-INPP5K fusion protein; MW = Molecular weight in kilo Dalton. **(B)** In live-imaged transfected COS-7 cells, the ER marker mCherry-Sec61β (red) colocalizes extensively with wild-type GFP-INPP5K (green). Images show a higher magnification of a detail of Figure 3A (left panel). Scale bars = 10 µm.

Figure S4. Muscle biopsies from individuals with bi-allelic *INPP5K* mutations.

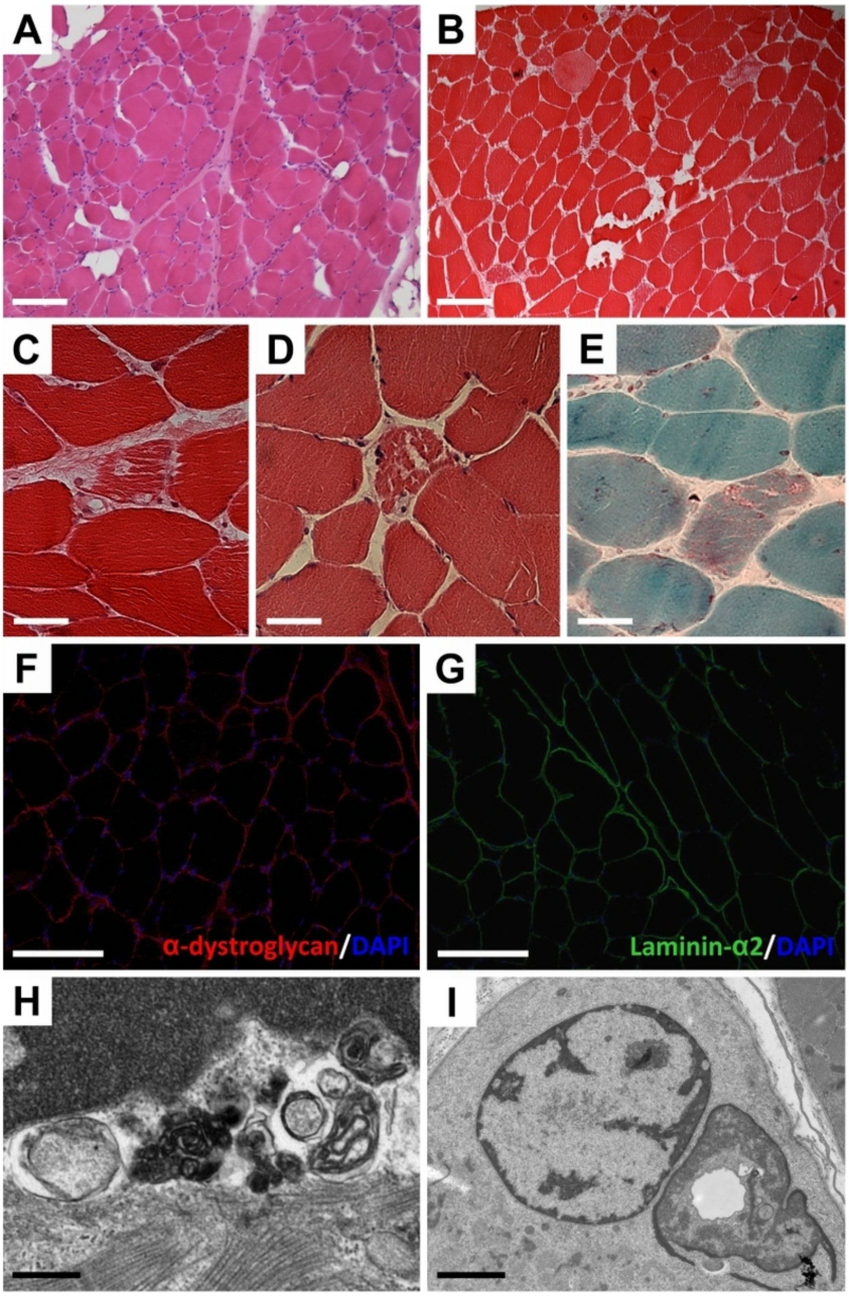

(A, B) H&E stain revealing variation in fiber size, rounding of fibers, increased endomysial collagen and some degree of fatty degeneration. **(C, D)** H&E stain, **(E)** Gomori trichrome stain showing vacuolated muscle fibers. With Gomori trichrome, some vacuoles contained red-stained material. **(F)** α-dystroglycan (clone VIA4-1) and **(G)** Laminin-α2 (clone 2H5) immunostainings gave positive results. **(H)** Electron micrograph showing a vacuole associated with myelin-like whorls and osmiophilic material. **(I)** Electron micrograph of nuclei in a degenerating muscle fiber, one with dense peripheral heterochromatin (left) and one partially surrounded by an electron-dense membranous structure (right). **(A)**: left M. vastus lateralis from individual IV.3, family A, taken at age 6 years, **(B-E, F, G)**: left M. biceps brachii from individual II.1, family H, taken at age 25 years), **(H, I)**: left quadriceps muscle from individual II.3, family B, taken at age 4 years). Scale bars **(A, B, F, G)** = 50 µm, **(C-E)** = 10 µm, **(H)** = 0.5 µm, **(I)** = 2 µm.

Figure S5. Levels of wild-type and mutant INPP5K in transfected cells.

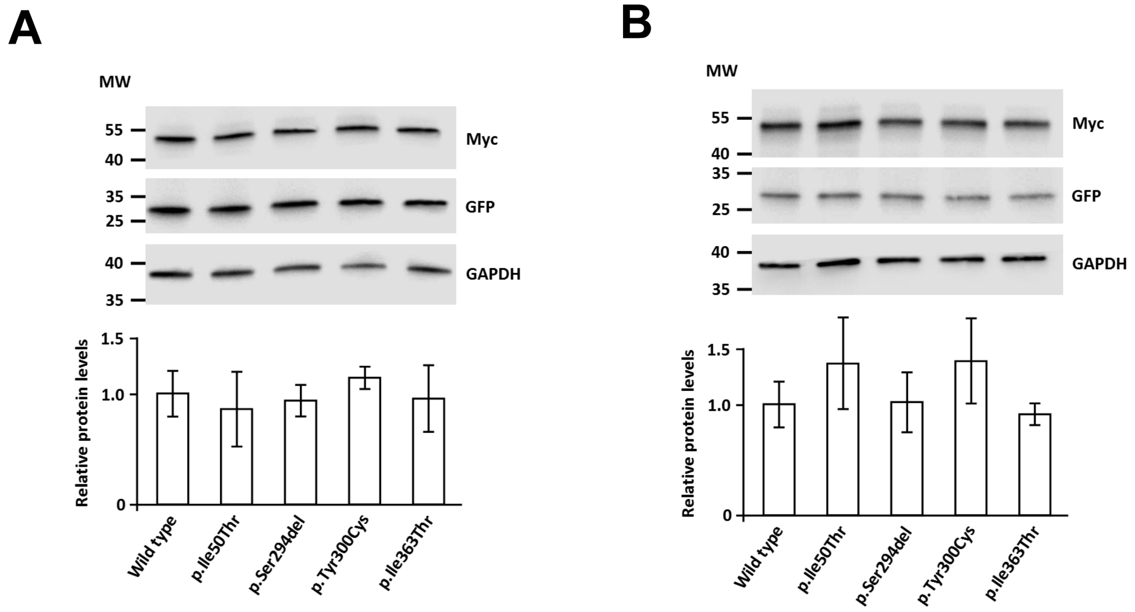

(A) Human *INPP5K* cDNA was amplified from human skeletal muscle mRNA and cloned in pCMV-Myc. INPP5K mutants were generated by site-directed mutagenesis and verified by sequencing. C2C12 cells were cultured in DMEM containing 10% fetal calf serum, 2 mM glutamine, 40 U/mL penicillin and 0.04 mg/mL streptomycin. Cells were cotransfected with Myc-tagged INPP5K expression constructs and an empty GFP expression plasmid (transfection efficiency control) using Amaxa Cell Line Nucleofector Kit V (Lonza). Cultured cells were lysed in cell lysis buffer (1% SDS, 10 mM Tris-HCl, pH 7.4) containing protease and phosphatase inhibitors. Protein preparations were incubated at 95°C for 5 min, debris was removed by 5 min centrifugation at 14,000 rpm at 4°C, and protein concentrations in the supernatants were measured with a BCA protein assay. Equal amounts of total protein were separated by SDS-polyacrylamide gel electrophoresis and transferred to nitrocellulose membranes. Membranes were blocked for 1 hr at RT in TBS-T with 5% non-fat milk and then incubated overnight at 4°C with primary antibodies mouse anti-Myc (Clontech; 1:1,000), rabbit anti-GFP (Abcam; 1:1,000) and rabbit anti-GAPDH (Merck Millipore; 1:500). Subsequently, blots were incubated with HRP-conjugated goat anti-mouse IgG and goat anti-rabbit IgG antibodies (both from Invitrogen; 1:5,000) for 1 hr at RT. Bands were detected with an ECL Detection Kit. Densitometry to quantify INPP5K levels was performed using ImageJ software. Signals obtained for INPP5K were normalized to GFP (transfection efficiency control) and GAPDH (loading control). Bars represent means of three independent experiments and error bars represent standard deviations. MW = Molecular weight in kilo Dalton. **(B)** Same analysis using COS-7 cells. Experimental conditions were identical to those described in (A) except for transfection of cells using JetPei Transfection Reagent (Peqlab) instead of nucleofection.

Figure S6. Akt phosphorylation in IGF-II-stimulated primary human skin fibroblasts.

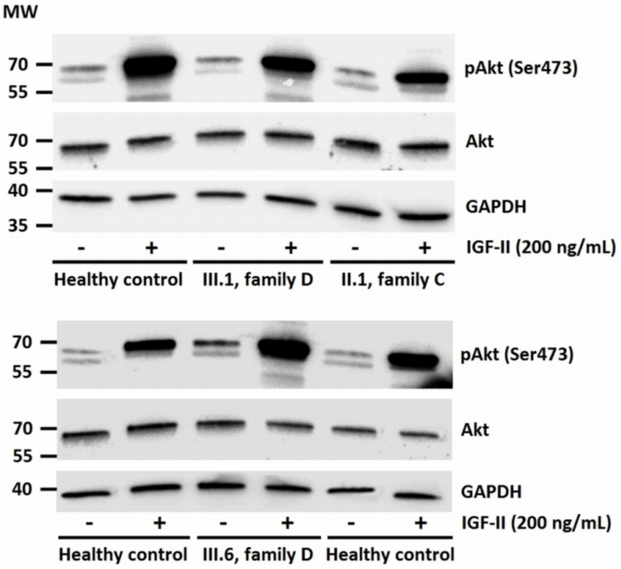

Skin fibroblasts from three individuals homozygous for the *INPP5K* mutation p.Ile50Thr (II.1, family C, III.1, family D and III.6, family D) and three healthy subjects were obtained from the MRC Centre for Neuromuscular Diseases Biobank, Newcastle, UK. Cells were cultured in DMEM containing 10% fetal calf serum, 2 mM glutamine, 40 U/mL penicillin and 0.04 mg/mL streptomycin. Prior to cell lysis, cultures were kept in serum-free DMEM for 48 hr and then stimulated with 200 ng/mL IGF-II (Merck Millipore) for 1 hr [2]. Cells were lysed in cell lysis buffer supplemented with protease and phosphatase inhibitors. Protein preparations were incubated at 95°C for 5 min, debris was removed by 5 min centrifugation at 14,000 rpm at 4°C, and protein concentrations in the supernatants were measured with a BCA protein assay. Equal amounts of total protein were run on SDS-polyacrylamide gels and blotted onto nitrocellulose membranes. Membranes were blocked for 1 hr at RT in TBS-T with 5% non-fat milk and then incubated overnight at 4°C with primary antibodies mouse anti-Akt, rabbit anti-phospho-Akt (Ser473) (both from Cell Signaling Technology; 1:2,000) and rabbit anti-GAPDH. Subsequently, blots were incubated with HRP-conjugated goat anti-mouse IgG and goat anti-rabbit IgG antibodies for 1 hr at RT. Bands were detected with an ECL Detection Kit. MW = Molecular weight in kilo Dalton.

Figure S7. MO-mediated double knockdown of *inpp5ka* and *inpp5kb* in zebrafish.

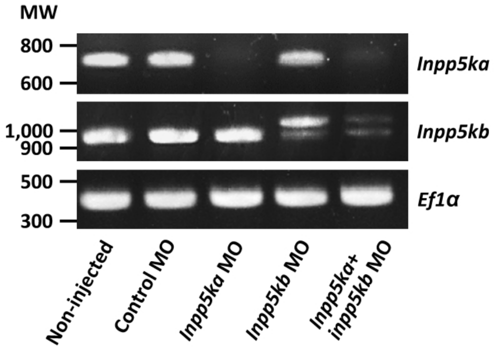

RT-PCR analysis of *inpp5ka* and *inpp5kb* transcripts from 48-hpf embryos injected with splice-blocking *inpp5ka* and *inpp5kb* MOs. RNA was isolated with Trizol reagent (Invitrogen). Reverse transcription was performed with the Superscript III First-Strand Synthesis System (Invitrogen). Target sequences were PCR-amplified using specific oligonucleotide primers, run on agarose gels and detected by SafeView Nucleic Acid Stain (NBS Biologicals). Top panel: Downregulation of *inpp5ka* in both the single and double knockdown was demonstrated with *inpp5ka*-specific PCR primers. Middle panel: Mis-spliced *inpp5kb* transcripts alongside small amount of retained wild-type transcript were detected in the single and double knockdown with *inpp5kb*-specific PCR primers. Bottom panel: *Ef1α* was used as an internal RT-PCR control to exclude variations in cDNA synthesis quality. MW = Molecular weight in base pairs.

Figure S8. Neuromuscular junction morphology in *inpp5ka+inpp5kb* morphants.

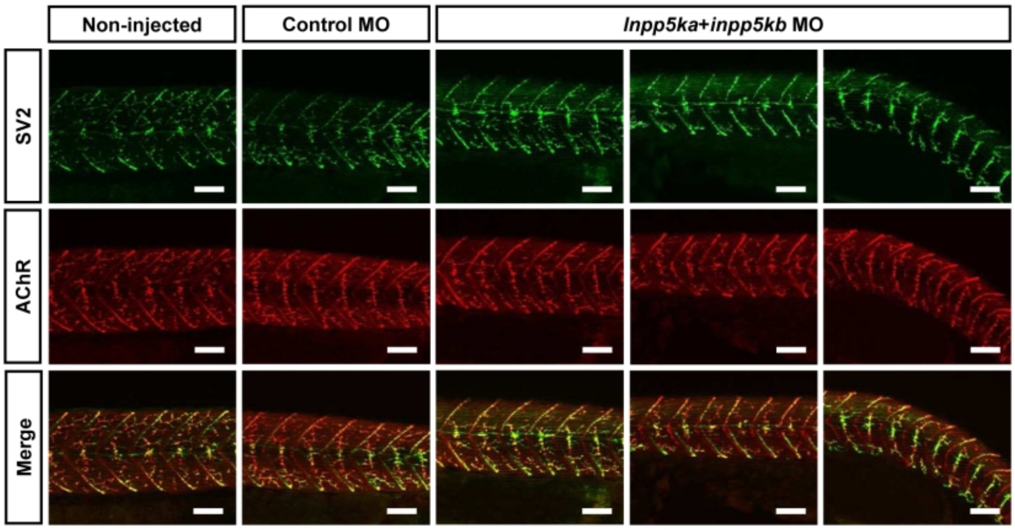

For whole-mount immunofluorescence staining, 48-hpf zebrafish embryos were fixed in 4% paraformaldehyde in PBS at 4°C overnight and blocked for 1 hr at RT in 5% horse serum in PBS, 0.1% Tween-20. Presynaptic motor nerve endings were visualized by incubation with mouse anti-synaptic vesicle protein 2 antibody (SV2, DSHB; 1:200) overnight at 4°C followed by Alexa Fluor 488-conjugated goat anti-mouse IgG antibody for 1 hr at RT. Acetylcholine receptors (AChR) were labelled with Alexa Fluor 594-conjugated α-bungarotoxin (Thermo Fisher Scientific; 1:1,000). Images were captured with a Nikon A1R confocal microscope (Nikon). Images for *inpp5ka+inpp5kb* morphants represent mild, moderate and severe phenotypes (from left to right) as staged macroscopically. Both in controls and in *inpp5ka+inpp5kb* morphants, motor axons had made branches into somites where they formed contacts with AChR clusters. Scale bars = 50 μm.

Figure S9. Quantification of ER stress response markers.

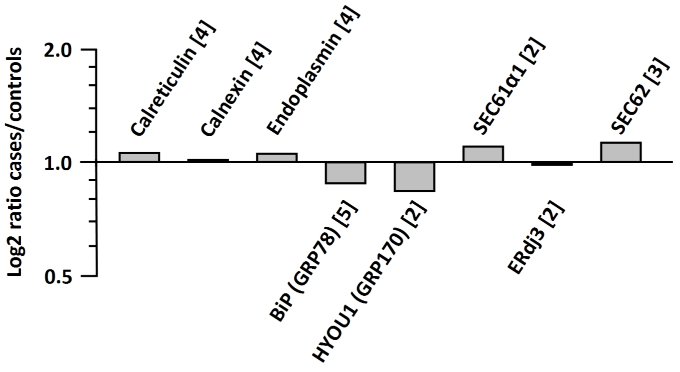

Skin fibroblasts from three individuals homozygous for the *INPP5K* mutation p.Ile50Thr (II.1, family C, III.1, family D and III.6, family D) and three healthy subjects were obtained from the MRC Centre for Neuromuscular Diseases Biobank, Newcastle, UK. Cells were cultured in DMEM containing 10% fetal calf serum, 2 mM glutamine, 40 U/mL penicillin and 0.04 mg/mL streptomycin. Cells were lysed in 1% SDS, 150 mM NaCl, 50 mM Tris-HCl, pH 7.8 containing protease and phosphatase inhibitors, and protein extracts were treated with benzonase (Merck Millipore) for 30 min at 37°C. Protein preparations were incubated at 95°C for 5 min, debris was removed by 30 min centrifugation at 18,000g at 4°C, and protein concentrations in the supernatants were measured with a BCA protein assay. Protein extracts were treated with 10 mM dithiothreitol for 30 min at 56°C followed by incubation with 30 mM iodoacetamide for 20 min at RT. Samples were digested with trypsin using filter-aided sample preparation [3-4] and then analyzed on an Ultimate 3000 nano RSLC system coupled to a Q Exactive HF mass spectrometer (Thermo Fisher Scientific) in parallel reaction monitoring mode. Peptides were pre-concentrated on a PepMap 100 µm x 2 cm C18 trapping column (Thermo Fisher Scientific) for 10 min using 0.1% trifluoroacetic acid at a flow rate of 20 µL/min, followed by separation on a PepMap 75 µm x 50 cm C18 main column (Thermo Fisher Scientific) with a 55 min gradient ranging from 5-42% of 84% acetonitrile, 0.1% formic acid at a flow rate of 250 nL/min. Mass spectra were acquired in the Orbitrap at a resolution of 30,000 with an automatic gain control target of 3 x 106, a maximum injection time of 100 ms, an isolation width of 0.4 m/z and a normalized collision energy of 27 in scheduled mode. Raw data was evaluated with Skyline software [5] and peak selection and assignment were manually validated. Unique peptides of proteins whose levels are altered under conditions of ER stress [6-7], calreticulin, calnexin, endoplasmin, BiP (GRP78), HYOU1 (GRP170), ERdj3, SEC61α1 and SEC62, were compared against an in-house spectral library. Peptide peak areas were used to determine median abundance of each protein of interest across all target protein-related peptides detected in two technical replicates of cases and controls. Median values were normalized to the median value calculated for proteome-wide peptides from the case or control group. Data are presented as the ratio cases median protein level/controls median protein level, plotted in logarithmic scale base 2. The counts of unique peptides for individual proteins are given in box brackets after the protein names.

Figure S10. Coprecipitation of BiP with INPP5K from transfected cells.

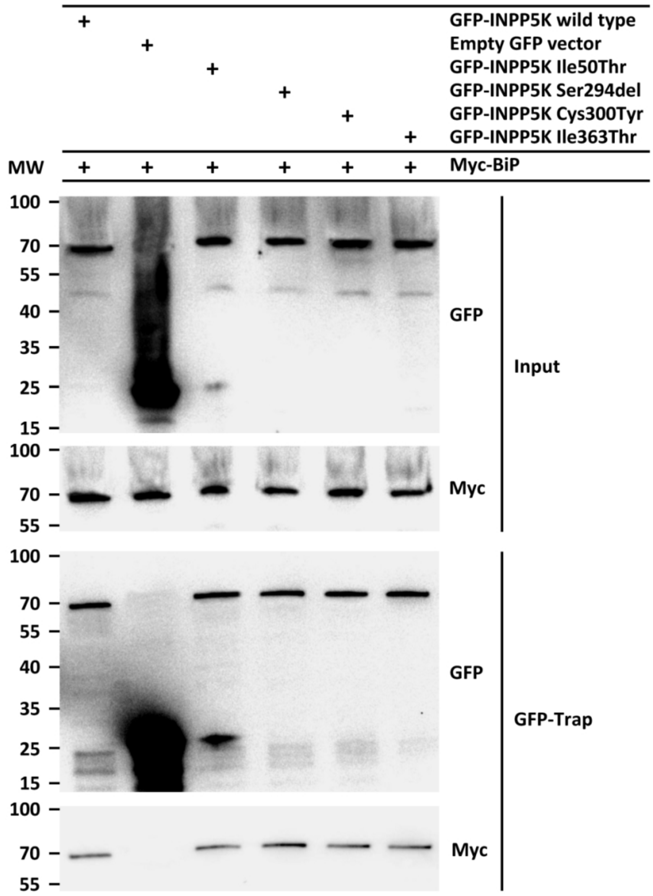

COS-7 cells were cotransfected with expression vectors for GFP-tagged INPP5K (or empty GFP vector) and Myc-tagged BiP (Addgene plasmid #27164, a gift from Ron Prywes [8]) using JetPei Transfection Reagent. Cells were lysed in 150 mM NaCl, 0.5 mM EDTA, 10 mM Tris-HCl, pH 7.5, 0.5% NP-40 supplemented with protease and phosphatase inhibitors. Protein extracts were incubated at 95°C for 5 min, debris was removed by 5 min centrifugation at 14,000 rpm at 4°C, and protein concentrations in the supernatants were measured with a BCA protein assay. Five hundred µg of total protein were mixed with GFP binding protein coupled to magnetic agarose beads (GFP-Trap [9]; ChromoTek) and left on a rotator for 1 hr at 4°C. Beads were magnetically separated, washed, resuspended in SDS sample buffer and boiled to elute bound proteins. Proteins were resolved by SDS-polyacrylamide gel electrophoresis, blotted onto PVDF membranes (Hybond; GE Healthcare) and transferred proteins were blocked for 1 hr at RT in TBS-T with 5% non-fat milk. To determine protein levels in transfected cells (Input) as well as amounts of precipitated GFP-INPP5K (or GFP alone) and bound Myc-BiP (GFP-Trap), membranes were incubated overnight at 4°C with primary antibodies mouse anti-Myc and rabbit anti-GFP followed by HRP-conjugated goat anti-mouse IgG and goat anti-rabbit IgG antibodies for 1 hr at RT. Bands were visualized with an ECL Detection Kit.

Figure S11. Measurement of autophagy in skeletal muscle and cultured skin fibroblasts.

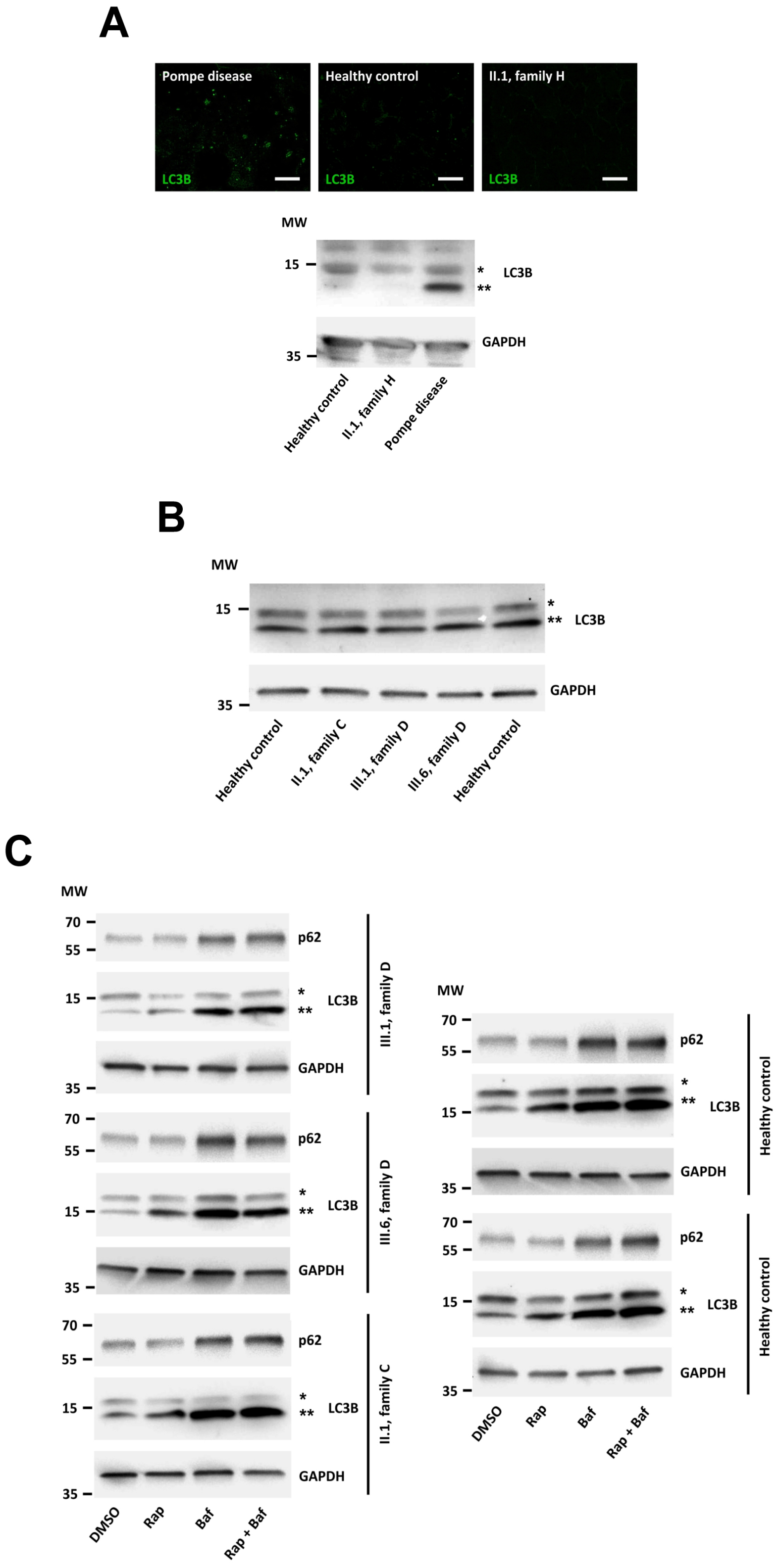

(A) Detection of LC3B in muscle biopsies from individual II.1, family H (homozygous *INPP5K* mutation p.Tyr300Cys), and healthy and pathological controls. Eight-µm cryostat sections of deep-frozen unfixed tissue samples were fixed with 4% paraformaldehyde, pH 7.4, for 10 min at RT, permeabilized with 0.1% Triton X-100 for 15 min and blocked in 5% horse serum in PBS for 1 hr at RT. Specimens were incubated at 4°C overnight with rabbit anti-LC3B antibody (Novus Biologicals; 1:100), followed (*continued on next page*)

(*legend to Figure S11, continued from previous page*) by Alexa Fluor 488-conjugated donkey anti-rabbit IgG antibody (Jackson ImmunoResearch; 1:500) for 1 hr at RT. Images were captured with a Zeiss Axiovert 200 M fluorescence microscope and a Zeiss AxioCam HR camera (Zeiss). For immunoblotting, skeletal muscle biopsy specimens were homogenized with a rotor-stator homogenizer in 1x PBS containing 1% SDS. Protein preparations were incubated at 95°C for 5 min, debris was removed by 5 min centrifugation at 14,000 rpm at 4°C, and protein concentrations in the supernatants were measured with a BCA protein assay. Equal amounts of total protein were separated on SDS-polyacrylamide gels and blotted onto PVDF membranes. Membranes were blocked for 1 hr at RT in TBS-T with 5% non-fat milk and then incubated overnight at 4°C with rabbit anti-LC3B (Novus Biologicals; 1:1,000) and rabbit anti-GAPDH antibodies. Subsequently, blots were incubated with HRP-conjugated goat anti-rabbit IgG antibody for 1 hr at RT. Bands were detected with an ECL Detection Kit. .* = LC3B-I, ** = LC3B-II. MW = Molecular weight in kilo Dalton. **(B)** Detection of LC3B in primary skin fibroblasts. Skin fibroblasts from three individuals homozygous for the *INPP5K* mutation p.Ile50Thr (II.1, family C, III.1, family D and III.6, family D) and two healthy subjects were obtained from the MRC Centre for Neuromuscular Diseases Biobank, Newcastle, UK. Cells were cultured in DMEM containing 10% fetal calf serum, 2 mM glutamine, 40 U/mL penicillin and 0.04 mg/mL streptomycin. Cells were lysed in radioimmunoprecipitation assay buffer (1% Triton X-100, 0.5% sodium deoxycholate, 1% SDS, 150 mM NaCl, 50 mM Tris-HCl, pH 7.4) containing protease and phosphatase inhibitors. Protein preparations were incubated at 95°C for 5 min, debris was removed by 5 min centrifugation at 14,000 rpm at 4°C, and protein concentrations in the supernatants were measured with a BCA protein assay. Equal amounts of total protein were run on SDS-polyacrylamide gels and transferred to PVDF membranes. Membranes were blocked for 1 hr at RT in TBS-T with 5% non-fat milk and then incubated overnight at 4°C with primary antibodies rabbit anti-LC3B and rabbit anti-GAPDH. Subsequently, blots were incubated with HRP-conjugated goat anti-rabbit IgG antibody for 1 hr at RT. Bands were detected with an ECL Detection Kit. MW = Molecular weight in kilo Dalton. **(C)** LC3B conversion and p62 levels in primary skin fibroblasts treated with autophagy modifiers. In addition to the procedures described in (B), cells were treated with 100 nM bafilomycin A1, 100 nM rapamycin or vehicle (DMSO) for 12 hr prior to cell lysis [10]. For detection of p62, a rabbit anti-p62 antibody (Sigma-Aldrich; 1:1,000) was used.

Table S1. Bioinformatic results for INPP5K amino acid substitutions.

Mutation	Mutation Taster prediction / score	PolyPhen-2 prediction / score	SIFT prediction / score	SNAP2 prediction / score	LRT prediction / LRT_{new} score	GERP++ score	PhyloP score
p.Ile50Thr	Disease causing / 1	Probably damaging / 0.999	Damaging / 0	Effect / 7	Deleterious / 1	5.4	1.062
p.Tyr300Cys	Disease causing / 1	Probably damaging / 1	Damaging / 0	Effect / 89	Deleterious / 1	5.53	0.991
p.Ile363Thr	Disease causing / 1	Probably damaging / 1	Damaging / 0	Effect / 22	Deleterious / 1	5.94	1.003

The score given by MutationTaster [11] is the probability of the prediction, i.e. a value close to 1 indicates a high "security" of the prediction. For PolyPhen-2 [12], scores near 1.0 are most strongly predicting a deleterious effect of an amino substitution. SIFT [13] scores ≤ 0.05 are assigned the prediction "damaging". SNAP2 [14] scores range from -100 strong neutral prediction to +100 strong effect prediction. Values for the LRT_{new} [15] score range from 0 to 1 with higher values indicating a variant is more likely to be deleterious. GERP++ [16] estimates evolutionary constraint of specific positions. Scores range from -12.3 to 6.17 with higher scores indicating more conserved sites. PhyloP [17] conservation scores are based on the multiple alignment of vertebrate genomes. Scores range from -5.172 to 1.062 and higher scores suggest stronger conservation of the site.

Supplementary References

1. Gudbjartsson, D.F., Jonasson, K., Frigge, M.L., and Kong, A. (2000). Allegro, a new computer program for multipoint linkage analysis. Nat Genet 25, 12-13.
2. Hartmann, W., Koch, A., Brune, H., Waha, A., Schüller, U., Dani, I., Denkhaus, D., Langmann., W., Bode, U., Wiestler, O.D., et al. (2005). Insulin-like growth factor II is involved in the proliferation control of medulloblastoma and its cerebellar precursor cells. Am J Pathol 166, 1153-1162.
3. Manza, L.L., Stamer, S.L., Ham, A.J., Codreanu, S.G., and Liebler, D.C. (2005). Sample preparation and digestion for proteomic analyses using spin filters. Proteomics 5, 1742-1745.
4. Wisniewski, J.R., Zougman, A., Nagaraj, N., and Mann, M. (2009). Universal sample preparation method for proteome analysis. Nat Methods 6, 359-362.
5. MacLean, B., Tomazela, D.M., Shulman, N., Chambers, M., Finney, G.L., Frewen, B., Kern, R., Tabb, D.L., Liebler, D.C., and MacCoss, M.J. (2010). Skyline: an open source document editor for creating and analyzing targeted proteomics experiments. Bioinformatics 26, 966-968.
6. Lee, A.S. (2014). Glucose-regulated proteins in cancer: molecular mechanisms and therapeutic potential. Nat Rev Cancer 14, 263-276.
7. Schroder, M., and Kaufman, R.J. (2005). The mammalian unfolded protein response. Annu Rev Biochem 74, 739-789.
8. Shen, J., Chen, X., Hendershot, L., and Prywes, R. (2002). ER stress regulation of ATF6 localization by dissociation of BiP/GRP78 binding and unmasking of Golgi localization signals. Dev Cell 3, 99-111.
9. Rothbauer, U., Zolghadr, K., Muyldermans, S., Schepers, A., Cardoso, M.C., and Leonhardt, H. (2008). A versatile nanotrap for biochemical and functional studies with fluorescent fusion proteins. Mol Cell Proteomics 7, 282-289.
10. Kimura, S., Fujita, N., Noda, T., and Yoshimori, T. (2009). Monitoring autophagy in mammalian cultured cells through the dynamics of LC3. Methods Enzymol 452, 1-12.
11. Schwarz, J.M., Rodelsperger, C., Schuelke, M., and Seelow, D. (2010). MutationTaster evaluates disease-causing potential of sequence alterations. Nat Methods 7, 575-576.
12. Adzhubei, I., Jordan, D.M., and Sunyaev, S.R. (2013). Predicting functional effect of human missense mutations using PolyPhen-2. Curr Protoc Hum Genet Chapter 7, Unit7 20.
13. Kumar, P., Henikoff, S., and Ng, P.C. (2009). Predicting the effects of coding non-synonymous variants on protein function using the SIFT algorithm. Nat Protoc 4, 1073-1081.
14. Hecht, M., Bromberg, Y., and Rost, B. (2015). Better prediction of functional effects for sequence variants. BMC Genomics 16 Suppl 8, S1.
15. Chun, S., and Fay, J.C. (2009). Identification of deleterious mutations within three human genomes. Genome Res 19, 1553-1561.
16. Davydov, E.V., Goode, D.L., Sirota, M., Cooper, G.M., Sidow, A., and Batzoglou, S. (2010). Identifying a high fraction of the human genome to be under selective constraint using GERP++. PLoS Comput Biol 6, e1001025.
17. Pollard, K.S., Hubisz, M.J., Rosenbloom, K.R., and Siepel, A. (2010). Detection of nonneutral substitution rates on mammalian phylogenies. Genome Res 20, 110-121.
