# Supplementary material for: Mutations in INPP5K, Encoding a Phosphoinositide 5-Phosphatase, Cause Congenital Muscular Dystrophy with Cataracts and Mild Cognitive Impairment
Source: Am J Hum Genet. 2017 Feb 9;100(3):523–36. doi: 10.1016/j.ajhg.2017.01.024 (PMC5339217; doi:10.1016/j.ajhg.2017.01.024)
Supplement: Document S2. Article plus Supplemental Data [file mmc2.pdf]

Mutations in *INPP5K*, Encoding a Phosphoinositide 5-Phosphatase, Cause Congenital Muscular Dystrophy with Cataracts and Mild Cognitive Impairment

Manuela Wiessner,[1,20] Andreas Roos,[2,3,20] Christopher J. Munn,[4,20] Ranjith Viswanathan,[1,21] Tamieka Whyte,[5,6] Dan Cox,[2] Benedikt Schoser,[1] Caroline Sewry,[5,7] Helen Roper,[8] Rahul Phadke,[5] Chiara Marini Bettolo,[2] Rita Barresi,[2,9] Richard Charlton,[2,9] Carsten G. Bönnemann,[10] Osório Abath Neto,[10] Umbertina C. Reed,[11] Edmar Zanoteli,[11] Cristiane Araújo Martins Moreno,[11] Birgit Ertl-Wagner,[12] Rolf Stucka,[1] Christian De Goede,[13,14] Tamiris Borges da Silva,[4] Denisa Hathazi,[3] Margherita Dell'Aica,[3] René P. Zahedi,[3] Simone Thiele,[1] Juliane Müller,[2] Helen Kingston,[15] Susanna Müller,[16] Elizabeth Curtis,[17] Maggie C. Walter,[1] Tim M. Strom,[18,19] Volker Straub,[2] Kate Bushby,[2] Francesco Muntoni,[5,6] Laura E. Swan,[4,20] Hanns Lochmüller,[2,20] and Jan Senderek[1,20,*]

Phosphoinositides are small phospholipids that control diverse cellular downstream signaling events. Their spatial and temporal availability is tightly regulated by a set of specific lipid kinases and phosphatases. Congenital muscular dystrophies are hereditary disorders characterized by hypotonia and weakness from birth with variable eye and central nervous system involvement. In individuals exhibiting congenital muscular dystrophy, early-onset cataracts, and mild intellectual disability but normal cranial magnetic resonance imaging, we identified bi-allelic mutations in *INPP5K*, encoding inositol polyphosphate-5-phosphatase K. Mutations impaired phosphatase activity toward the phosphoinositide phosphatidylinositol (4,5)-bisphosphate or altered the subcellular localization of INPP5K. Downregulation of INPP5K orthologs in zebrafish embryos disrupted muscle fiber morphology and resulted in abnormal eye development. These data link congenital muscular dystrophies to defective phosphoinositide 5-phosphatase activity that is becoming increasingly recognized for its role in mediating pivotal cellular mechanisms contributing to disease.

Introduction

Congenital muscular dystrophies (CMDs) are clinically and genetically heterogeneous inherited disorders in which muscle weakness typically manifests at birth or in infancy.[1] Delayed motor milestones, poor motor abilities, and joint or spinal rigidity are often the presenting features. Muscle weakness may remain stable or deteriorate over time. Complications include contractures, spinal deformities, and respiratory compromise. Cardiac involvement, cognitive impairment, white matter and structural abnormalities of the brain, seizures, and eye abnormalities may occur depending on the genetic cause. For example, early cataracts, cerebellar atrophy, and variable intellectual disability suggest Marinesco-Sjögren syndrome (MSS [MIM: 248800]), which also features characteristic ultrastructural muscle pathology (dense perinuclear membranous structures).[2–4] Most CMDs are inherited in an autosomal-recessive manner with the exception of de novo dominant inheritance in CMDs caused by *LMNA* mutations (MIM: 613205) and some cases of Ullrich CMD (MIM: 254090). Mutations occur in genes encoding structural proteins of the extracellular matrix, enzymes catalyzing protein glycosylation, and proteins of the endoplasmic reticulum (ER) and nuclear envelope.[5] Mutations can be identified in 25%–50% of CMD-affected case subjects,[6] suggesting the existence of unidentified additional genes harboring mutations causing CMD and underscoring the need for ongoing investigation into the genetic causes of CMD.

[1]Friedrich-Baur-Institute, Department of Neurology, Ludwig Maximilians University Munich, 80336 Munich, Germany; [2]John Walton Muscular Dystrophy Research Centre, MRC Centre for Neuromuscular Diseases, Institute of Genetic Medicine, Newcastle University, Newcastle upon Tyne NE1 3BZ, UK; [3]Leibniz-Institut für Analytische Wissenschaften (ISAS), 44227 Dortmund, Germany; [4]Department of Cellular and Molecular Physiology, Institute of Translational Medicine, University of Liverpool, Crown Street, Liverpool L69 3BX, UK; [5]UCL Great Ormond Street Institute of Child Health & Great Ormond Street Hospital for Children, London WC1N 1EH, UK; [6]MRC Centre for Neuromuscular Diseases, UCL Institute of Neurology, London WC1N 3BG, UK; [7]Wolfson Centre for Inherited Neuromuscular Disorders, RJAH Orthopaedic Hospital, Oswestry SY10 7AG, UK; [8]Birmingham Heartlands Hospital, Heart of England NHS Foundation Trust, Birmingham B9 5SS, UK; [9]Rare Diseases Advisory Group Service for Neuromuscular Diseases, Newcastle upon Tyne Hospitals NHS Foundation Trust, Newcastle upon Tyne NE1 3BZ, UK; [10]Neuromuscular and Neurogenetic Disorders of Childhood Section, Neurogenetics Branch, National Institute of Neurological Disorders and Stroke, NIH, Bethesda, MD 20814, USA; [11]Departamento de Neurologia, Faculdade de Medicina da Universidade de São Paulo, 01246-903 São Paulo, Brazil; [12]Institute for Clinical Radiology, Ludwig Maximilians University Munich, 81377 Munich, Germany; [13]Department of Paediatric Neurology, Royal Preston Hospital, Lancashire Teaching Hospitals NHS Foundation Trust, Preston PR2 9HT, UK; [14]Faculty of Health and Medicine, Lancaster University, Lancaster LA1 4YG, UK; [15]Manchester Centre for Genomic Medicine, Central Manchester University Hospitals NHS Foundation Trust, Saint Mary's Hospital, Oxford Road, Manchester M13 9WL, UK; [16]Institute of Pathology, Ludwig-Maximilians University Munich, 80337 Munich, Germany; [17]Department of Cellular Pathology, Queen Elizabeth Hospital Birmingham, University Hospitals Birmingham NHS Foundation Trust, Birmingham B15 2TH, UK; [18]Institute of Human Genetics, Helmholtz Zentrum München, 85764 Neuherberg, Germany; [19]Institute of Human Genetics, Technische Universität München, 81675 Munich, Germany
[20]These authors contributed equally to this work
[21]Present address: Developmental Biology Unit, EMBL Heidelberg, 69117 Heidelberg, Germany
*Correspondence: jan.senderek@med.uni-muenchen.de
http://dx.doi.org/10.1016/j.ajhg.2017.01.024.
© 2017 The Author(s). This is an open access article under the CC BY license (http://creativecommons.org/licenses/by/4.0/).

Phosphatidylinositol (PtdIns) is a membrane phospholipid that can be reversibly phosphorylated at the 3, 4, and 5 positions of the inositol ring. Resulting phosphoinositides provide the basis for the synthesis of the second messengers diacylglycerol and inositol (1,4,5)-trisphosphate (Ins(1,4,5)P_3, IP_3), which mobilize intracellular calcium and activate protein kinase C.[7] Moreover, through interactions between their phosphorylated head groups and protein modules, phosphoinositides recruit proteins to the cytosolic leaflet of membrane bilayers where they regulate multiple processes including the assembly of signaling scaffolds, biogenesis of transport vesicles, endocytosis and secretion, actin nucleation, microtubule dynamics, and transport of ions and metabolites.[8–11] The spatial and temporal control of the presence of each phosphoinositide at plasma and organelle membranes[12] is ensured by the action of kinases and phosphatases that are differentially located on specific membranes.[13] The relevance of proper phosphoinositide metabolism for cell and organ function is emphasized by the growing number of human diseases resulting from mutations in genes encoding enzymes that catalyze interconversion from one phosphoinositide to another.[14] While such mutations have been identified in other inherited neurological and neuromuscular disorders, including oculocerebrorenal syndrome of Lowe (MIM: 309000),[15] lethal congenital contractural syndrome type 3 (MIM: 611369),[16] X-linked myotubular myopathy (MIM: 310400),[17] and hereditary polyneuropathies (MIM: 601382, 604563, 611228),[18–21] no phosphoinositide metabolizing enzymes have been directly linked to CMD. Here we report that mutations in the gene encoding inositol polyphosphate-5-phosphatase K, INPP5K, cause a distinct form of CMD.

Material and Methods

Study Participants

The study population included 64 index case subjects and additional affected and unaffected family members who were referred to our laboratory for *SIL1* (MIM: 608005) mutation screening for suspected MSS. None of these individuals carried heterozygous or bi-allelic *SIL1* variants with predicted or known pathogenicity. Thirty-five affected individuals presented with both early-onset cataracts and skeletal muscular disease and occasionally one or several additional symptoms or signs (most commonly ataxia, cerebellar atrophy, hypotonia, motor delay, intellectual disability, somatic growth retardation, spasticity, seizures, and microcephaly). Twenty-nine index case subjects exhibited only one of either early cataracts or skeletal muscular disease, always combined with one or more aforementioned additional symptoms and signs. The study was conducted in accordance with national legislations and was approved by institutional review boards in Munich, London, Newcastle, and at the NIH. Informed consent was obtained from the probands or their legal guardians.

Reagents

If not stated otherwise, reagents were obtained from Sigma-Aldrich. Sequences of oligonucleotide primers (Metabion) used in this study are available upon request.

Whole-Exome Sequencing

The index case subject of family A (II.3) underwent whole-exome sequencing on a Genome Analyzer HiSeq 2000 system (Illumina) after in-solution enrichment of exon and flanking intron sequences (SureSelect Human all Exon 50 Mb kit v4; Agilent) and indexing of samples for multiplex-sequencing (Multiplexing Sample Preparation Oligonucleotide Kit; Illumina). Read alignment was performed with BWA v.0.5.8 to the human genome assembly hg19. Single-nucleotide variants and small insertions and deletions were called with SAMtools v.0.1.7. Variant annotation was performed with custom Perl scripts, integrating data from dbSNP135 and the UCSC Genome Browser Known Genes track. We excluded all nongenic, intronic (other than canonical splice sites), and synonymous variants, HapMap single-nucleotide polymorphisms (SNPs) present in dbSNP135 with an average heterozygosity greater than 0.02, and variants present in >15 of >7,000 in-house exomes from individuals with unrelated diseases. Next, because a recessive disease model and a common ancestral allele (based on parental consanguinity) were expected, we gave priority to homozygous variants.

Mutation Detection in Additional Families

The coding exons and flanking intron regions of *INPP5K* (GenBank: NM_016532.3) were searched for mutations by Sanger sequencing of DNA samples obtained from additional index case subjects included in the study population. Oligonucleotide primers for PCR amplification and sequencing were designed using Primer3 software based on the Human Genome Browser genomic sequence of *INPP5K*. PCR products were sequenced using the BigDye Terminator v.3.1 Ready Reaction Cycle Sequencing Kit (Applied Biosystems) and capillary electrophoresis and detection on a 3730 DNA Analyzer (Applied Biosystems).

Expression Constructs

Human *INPP5K* cDNA was amplified from human skeletal muscle mRNA and cloned in pAcGFP-C1 (for mammalian expression) and pGEX-4T-2 (for bacterial expression). INPP5K mutants were generated by site-directed mutagenesis[22] and verified by sequencing. The expression vector for the ER marker mCherry-Sec61β (Addgene plasmid #49155) was a gift from Gia Voeltz.[23]

Cell Culture and Transfection

COS-7 cells were cultured in Dulbecco's Modified Eagle's medium (DMEM) containing 10% fetal calf serum, 2 mM glutamine, 40 U/mL penicillin, and 0.04 mg/mL streptomycin. Transfection of COS-7 cells for live cell imaging was performed on 80% confluent cells plated on 35-mm glass bottomed dishes (MatTek) using Lipofectamine 2000 (Invitrogen) and constructs for GFP-INPP5K and mCherry-Sec61β.

Fluorescence Microscopy

Live COS-7 cells cotransfected with GFP-tagged wild-type and mutant INPP5K and mCherry-Sec61β constructs were examined by fluorescence microscopy using a 3i-Marianas spinning disc confocal microscope (Intelligent Imaging Innovations). All constructs were transfected and imaged simultaneously in a single experiment. Ten to 15 random image fields were acquired at 40× magnification for each construct and 60–120 transfected cells per construct were assigned to one of three phenotypes by an investigator blind to the *INPP5K* genotype: reticular ER-like staining, punctate or partial ER-like staining, and diffuse cytosolic

staining. For whole-mount immunofluorescence staining, zebrafish embryos were fixed in 4% paraformaldehyde in phosphate-buffered saline (PBS) at 4°C overnight and blocked for 1 hr at room temperature (RT) in 5% horse serum in PBS, 0.1% Tween-20 before incubation overnight at 4°C with primary antibodies mouse anti-slow muscle myosin heavy chain (slow MyHc, clone F59, Developmental Studies Hybridoma Bank [DSHB]; 1:50) and mouse anti-fast muscle myosin heavy chain (fast MyHc, clone F310, DSHB; 1:200). Incubation with secondary antibody Alexa Fluor 488-conjugated goat anti-mouse immunoglobulin G (IgG) (Thermo Fisher Scientific; 1:200) was performed for 1 hr at RT. Images were captured with a Nikon A1R confocal microscope (Nikon).

Measurement of INPP5K Phosphatase Activity

Wild-type and mutant GST-tagged full-length INPP5K was expressed in BL21 pLysS cells and purified on GSA beads (Thermo Fisher Scientific) in assay buffer (50 mM Tris-HCl [pH 7.5], 150 mM NaCl, 10 mM $MgCl_2$) plus 1% Triton X-100 and EDTA-free protease inhibitors (Roche Diagnostics). After washing, aliquots of beads were run on Coomassie gels to determine the abundance of full-length fusion proteins. Beads bearing equal amounts of fusion proteins were incubated in assay buffer containing 135 μM PtdIns(4,5)P_2diC8, and free phosphate was measured using the Malachite Green assay kit (Echelon Biosciences). Results of three independent experiments were presented as mean ± standard deviation. To minimize variability between purifications, all constructs were freshly prepared and purified in parallel for each experiment, and beads used in the assay were afterward run on Coomassie gels to confirm equal protein loading.

Structural Model of INPP5K

We modeled INPP5K structure by threading INPP5K sequence on the closest available orthologous crystal structures, OCRL (catalytic domain, PDB: 4CMN) and NDP52 (SKICH domain, PDB: 3VVW), using the Phyre2 server.[24]

Zebrafish Husbandry and Observation

We used the golden strain (*slc24a5b1/+*) of zebrafish (Zebrafish International Resource Center). Larvae were raised and staged as described.[25] Video recordings of embryos were captured using a CMLN-13S2M camera (ClearView Imaging) mounted to a Leica stereomicroscope (Leica). Light microscopy images were taken with a Leica dissection stereomicroscope equipped with a DFC 420C Leica digital camera (Leica). Touch-evoked swimming response was elicited by touching the embryos with a pipette tip. Measurements of the eye diameter and the head diameter (dorsal-ventral axis) were taken and a ratio between the two was calculated. One-tailed Student's t test was used to assess statistical significance.

Antisense Morpholino Oligonucleotide Knockdown

Antisense morpholino oligonucleotides (MOs) were purchased from Gene Tools. The MOs were designed based on the sequence of the zebrafish *INPP5K* orthologs *inpp5ka* (GenBank: XM_005157623.3) and *inpp5kb* (GenBank: XM_005155275.3). We established splice-blocking MOs directed against the splice donor site of intron 4 of *inpp5ka* (5′-CAGACTGAAGAGGAGCAG CATTCAA-3′) and against the splice donor site of intron 4 of *inpp5kb* (5′-TAGACTGGGACACATTTGCTCAGGT-3′). The Gene Tools standard control MO (5′-CCTCTTACCTCAGTTACAATT TATA-3′) was used as a negative control for the effects of MO injections. Embryos were injected with control MO (5 ng) or both anti-INPP5K MOs (2.5 ng of *inpp5ka* MO, 5 ng of *inpp5kb* MO) and efficient gene knockdown was verified by RT-PCR. 29% of non-injected embryos, 37% of embryos injected with control MO, and 64% of *inpp5ka*+*inpp5kb* double-knockdown morphants were dead. Five independent MO injection experiments were performed for each MO and at least 500 injected embryos were evaluated for each MO.

Results

Bi-allelic Mutations in *INPP5K* Are Associated with CMD with Early-Onset Cataracts

A consanguineous Bangladeshi multiplex family with two affected and four unaffected children (family A; Figure 1A) presented the possibility of locating the disease locus by linkage analysis. Genome-wide genotyping with approximately 300,000 SNP markers revealed two hits on chr10q11.22–10q21.1 and chr17p13.3–17p13.2 with a maximum LOD score of 2.27 (Figure S1). The 5.58-Mb region of interest on the short arm of chr17 was further supported by the results of short tandem repeat marker genotyping in 12 out of 43 additional families suitable for haplotype analysis. A subgroup of these families was also tested for the potential chr10 locus; 1 out of 22 families was compatible with localization of so far unknown CMD mutations on chr10 (data not shown).

We next performed whole-exome sequencing on the index case subject of family A. The average read depth was 129 with 94% of the targeted regions covered at least 20-fold. After read alignment, variant calling, annotation, and filtering, we focused our analysis on non-synonymous homozygous variants located in the regions of interest on chr10q and chr17p and reduced potential disease-causing variants to two changes on chr17p. A c.149T>C (p.Ile50Thr) variant in *INPP5K* (GenBank: NM_016532.3) (Figures 1A and S2) was a non-conservative change, affected a strictly conserved amino acid, was predicted to interfere with normal protein function by bioinformatic algorithms (Table S1), and was absent from databases (dbSNP146, Exome Aggregation Consortium [ExAC], and >7,000 in-house exomes). The second variant, c.1156C>G (p.Leu386Val) in *SLC52A1* (GenBank: NM_017986.3), was considered unlikely as it resulted in a conservative change of a non-conserved amino acid, was predicted benign by bioinformatic algorithms, and had a MAF of up to 0.5% in ExAC subpopulations.

We obtained further evidence for a causative role of *INPP5K* mutations when extending mutation screening to the 12 families homozygous for the chr17p locus or compatible with linkage to this region. Sanger sequencing of the *INPP5K* coding region yielded bi-allelic mutations in six pedigrees (families B–G, Figures 1A and S2). Sanger sequencing of 21 isolated case subjects with non-consanguineous parents resulted in the identification of one individual carrying bi-allelic *INPP5K* mutations (family H,

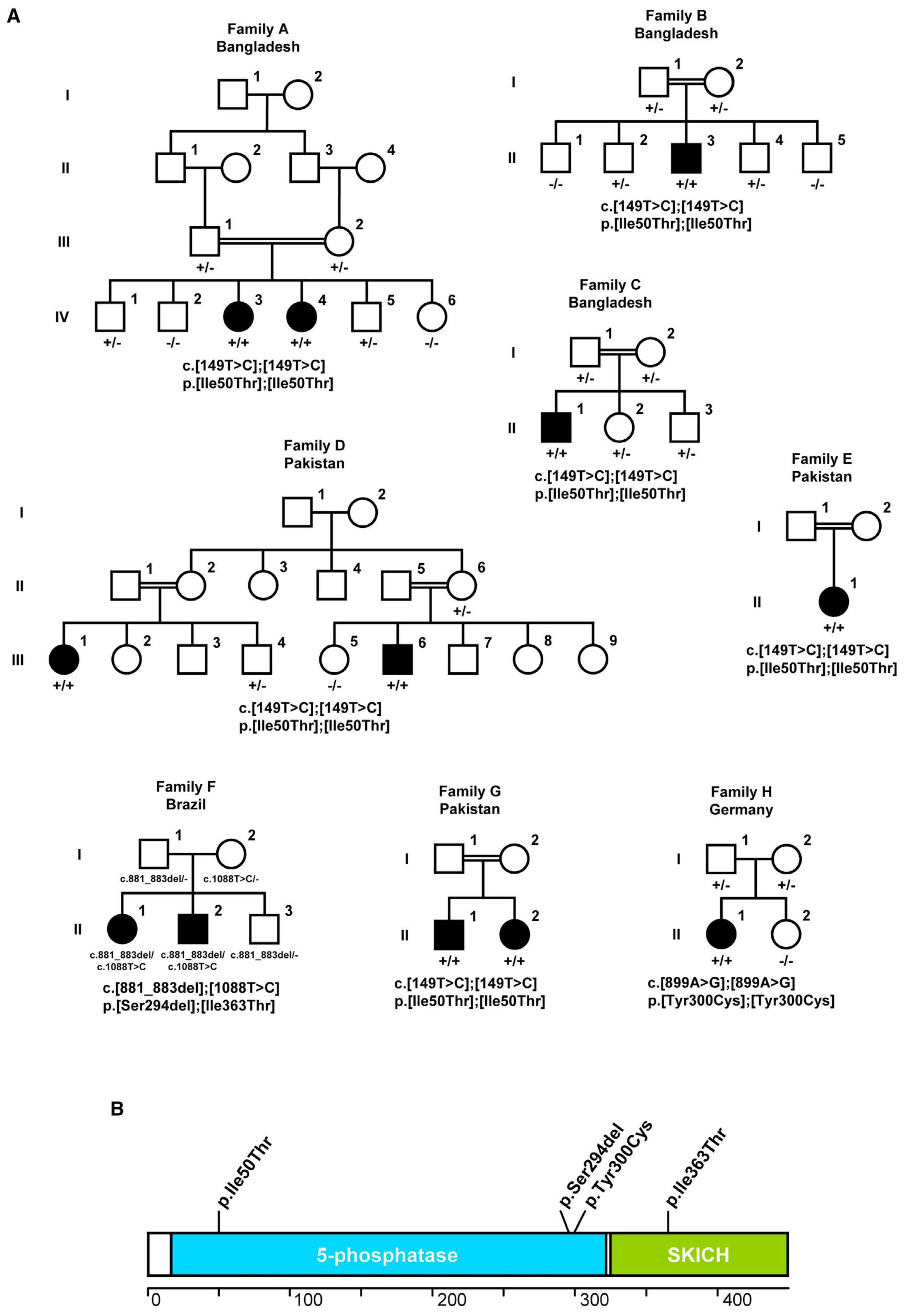

Figure 1. ***INPP5K*** **Mutations in Families with CMD and Cataracts**
(A) Pedigrees of families with identified homozygous or compound heterozygous *INPP5K* mutations. Squares represent males and circles represent females. Filled symbols represent affected individuals. *INPP5K* genotypes of individuals from whom a DNA sample was available are given below the pedigree symbols. Unaffected individuals were either heterozygous carriers or homozygous for the wild-type allele. +/+ indicates homozygous for mutation; +/− indicates heterozygous; −/− indicates homozygous for wild-type.
(B) Schematic representation of INPP5K and distribution of mutations. Amino acid numbering is shown below. Abbrevaitions: 5-phosphatase, inositol 5-phosphatase domain; SKICH, skeletal muscle and kidney-enriched inositol phosphatase carboxyl homology domain.

Figures 1A and S2). Altogether, we found four different *INPP5K* mutations consisting of three missense mutations (c.149T>C [p.Ile50Thr], c.899A>G [p.Tyr300Cys], and c.1088T>C [p.Ile363Thr]) and one in-frame deletion (c.881_883delCCT [p.Ser294del]) (Figure 1B). Whenever DNA from family members was available, we observed that the disease segregated with recessive inheritance of the *INPP5K* mutations (Figure 1A).

In keeping with *INPP5K* mutations causing a condition affecting skeletal muscle and eye, we confirmed presence of mouse INPP5K in these tissues (Figure S3A). *INPP5K* is a 12-exon gene encoding a 448-amino acid protein containing a 5-phosphatase domain.[26] An additional C-terminal SKICH motif has been linked to targeting of INPP5K to the ER membrane;[27] indeed, we observed colocalization of INPP5K with ER membranes in COS-7 cells cotransfected with GFP-INPP5K and the ER membrane marker mCherry-Sec61β (Figure S3B). The three missense mutations identified in our study affected strictly conserved amino acids and were predicted as deleterious to protein function or disease causing by 5/5 bioinformatic algorithms (Table S1). The c.149T>C (p.Ile50Thr) and c.1088T>C (p.Ile363Thr) variants were not observed in public databases (dbSNP146, ExAC) or in in-house exome datasets, which altogether allow interrogation of exome data of about 70,000 individuals. For the c.899A>G (p.Tyr300Cys) and c.881_883delCCT (p.Ser294del) variants, there were single heterozygous samples among the 60,000 individuals in ExAC (MAF 0.0000082; highest observed MAF in a subpopulation 0.00012 [East Asians] for p.Tyr300Cys and 0.00015 [Finnish Europeans] for p.Ser294del). Both of these samples probably represent carriers unaffected by *INPP5K* mutation-associated disease.

INPP5K Mutations Result in a Consistent and Recognizable Phenotype

All individuals with identified bi-allelic *INPP5K* mutations presented with a homogeneous clinical picture including both CMD and early-onset cataracts, usually with mild intellectual disability. The cerebellum appeared normal (Table 1, Figures 2A–2C). Notably, linkage to chr17 or *INPP5K* mutations were excluded in 29 families and isolated case subjects presenting with an incomplete phenotype, i.e., either skeletal muscular disease or early cataracts together with variable additional clinical manifestations (mainly polyneuropathy, cerebellar atrophy, or other brain malformations). Detection of *INPP5K* mutations in only 8 out of 35 families and index case subjects with combined skeletal muscular disease and early-onset cataracts might be explained by *INPPK* variants that are missed by commonly applied diagnostic strategies (e.g., genomic rearrangements or small deep intronic or regulatory variants), alternative effects of synonymous variants or locus heterogeneity.

The presenting symptoms were early bilateral lens opacities, muscle weakness from birth, motor or global developmental delay, or abnormal gait noted soon after independent walking had been acquired. Although affected individuals usually reached their motor milestones late, they all eventually acquired independent walking and muscle weakness, which was generally most prominent in proximal lower limb muscles, stabilized for several years. During the course of the disease, motor capabilities appeared to deteriorate, and (except for family A) older cases (families D, F, H) had lost ambulation (Table 1). Five subjects developed respiratory compromise. No facial or oculomotor weakness or cardiac involvement was observed. Creatine kinase values were invariably markedly elevated (mean ×7 of the upper normal limit, range ×3 to ×14). Electromyography (EMG) results in three affected individuals were in accordance with a myopathic process while motor nerve conduction studies did not demonstrate substantial abnormalities. Muscle magnetic resonance imaging (MRI) in individual II.1, family H revealed progressive degenerative myopathy (Figures 2D and 2E). Cognitive deficits, usually mild, were recorded in eight probands while four individuals had normal intelligence. A few affected individuals had additional symptoms including contractures, scoliosis, spinal rigidity, microcephaly, hyperlaxity in finger joints, intention tremor, seizures, or hypogonadism. No white matter and structural abnormalities of the brain were recorded except for II.1 (family H) who had widened inner and outer cerebrospinal liquor spaces but no focal alteration (Figure 2C) along with mild cognitive impairment.

Muscle biopsies had been taken previously from nine individuals with *INPP5K* mutations for diagnostic purposes (Table 1). Muscle pathology was largely nonspecific, showing variable degrees of dystrophic features, including increased range of muscle fiber size with small and hypertrophic fibers, muscle fibrosis, excess adipose tissue, occasional fibers with internal nuclei, rare necrotic fibers, and a few basophilic (regenerating) fibers (Figures 2F, S4A, and S4B). There were no abnormalities of blood vessels, inflammatory changes, or group atrophy. Three biopsies showed variable numbers of vacuolated muscle fibers (Figures S4C–S4E). Some vacuoles presented with increased acid phosphatase and non-specific esterase activity or as rimmed vacuoles (Figure S4E). The distribution of fiber types was sometimes uneven with several fascicles displaying a type 1 or type 2 predominance. Oxidative enzyme localization was generally normal with no or only rare areas devoid of activity. No inclusions or excess storage material were seen. Immunohistochemistry for dystrophin, sarcoglycans, α-dystroglycan, and laminin-α2 (merosin) was normal (Figures S4F and S4G). In some biopsies, cytoplasmic labeling for α-B crystallin, VCP, and p62 as well as developmental and fetal myosin was observed in a variable proportion of fibers. Electron microscopy was performed on two biopsies (II.3 from family B and II.1 from family H) and revealed several fibers with pronounced reduction of myofibrils and disrupted Z line material. Electron microscopy further confirmed the presence of occasional small vacuoles (Figure S4H), sometimes filled with

Table 1. Clinical Features of Individuals with Bi-allelic *INPP5K* Mutations

Family	Subject	Sex	Age	Ethnic Origin	Consanguinity	*INPP5K* Variant	Initial Presenting Symptom (at Age)	Cataracts (Age at Diagnosis)	Hypotonia	Delayed Motor Milestones
A	IV.3	F	21 y	Bangladesh	+	p.[Ile50Thr];[Ile50Thr]	motor developmental delay (15 m)	+ (6 y)	+	+
	IV.4	F	22 y				motor developmental delay (15 m)	+ (4 y)	+	+
B	II.3	M	8.5 y	Bangladesh	+	p.[Ile50Thr];[Ile50Thr]	global developmental delay (10 m)	+ (12 m)	+	+
C	II.1	M	11 y	Bangladesh	+	p.[Ile50Thr];[Ile50Thr]	cataracts (4 y)	+ (4 y)	−	−
D	III.1	F	35 y	Pakistan	−	p.[Ile50Thr];[Ile50Thr]	walking difficulty (2 y)	+ (3 y)	+	+
	III.6	M	25 y				cataracts (1.5 y)	+ (1.5 y)	+	−
E	II.1	F	9.5 y	Pakistan	+	p.[Ile50Thr];[Ile50Thr]	global developmental delay (2.5 y)	+ (5 y)	+	+
F	II.1	F	37 y	Brazil	−	p.[Ser294del];[Ile363Thr]	hypotonia (birth)	+ (6 m)	+	+
	II.2	M	35 y				hypotonia (birth)	+ (6 m)	+	+
G	II.1	M	7 y	Pakistan	+	p.[Ile50Thr];[Ile50Thr]	hypotonia, motor delay (birth)	+ (early childhood)	+	+
	II.2	F	6 y				hypotonia, motor delay (birth)	+ (early childhood)	+	+
H	II.1	F	41 y	Germany	−	p.[Tyr300Cys];[Tyr300Cys]	motor developmental delay (1 y)	+ (5 y)	+	+

Abbreviations are as follows: F, female; M, male; m, month(s); y, year(s); +, present; −, not present; NA, not available; ND, not determined; CK, serum creatine kinase; P > D, proximal muscles more severely affected than distal muscles; LL > UL, lower limb muscles more severely affected than upper limb muscles. Best motor ability: 0, normal walking, running, and jumping; 1, normal walking, no running and jumping; 2, walks longer distances unsupported, abnormal gait; 3, walks a few steps unsupported, requires walking aids or wheelchair for longer distances; 4, walks only with walking aids or uses wheelchair most of the time; 5, wheelchair bound.

myelin-like whorls as seen in rimmed vacuoles. In one of the two biopsies studied by electron microscopy (II.3, family B), several myonuclei had dense peripheral heterochromatin or were filled with osmiophilic material. Some of these abnormal nuclei also appeared to have an incomplete nuclear membrane or were surrounded by a dense membrane-like structure (Figure S4I).

INPP5K Mutants Alter Subcellular Localization or Impair Phosphatase Activity for PtdIns(4,5)P_2

Immunoblotting of protein preparations from C2C12 and COS-7 cells transfected with expression vectors for INPP5K wild-type and mutants showed no major effect of mutations on protein levels (Figures S5A and S5B). The mutants p.Ile50Thr, p.Ser294del, and p.Tyr300Cys showed complete or partial perinuclear localization when overexpressed as GFP-fusion proteins in COS-7 cells (Figure 3A), largely indistinguishable from the wild-type protein, which is regularly located at the ER (Figures 3A and S3B and Gurung et al.[27]). The p.Ile363Thr mutant affecting the SKICH motif, however, displayed a striking tendency for a more diffuse distribution in transfected cells (Figure 3A).

INPP5K has been reported to dephosphorylate both PtdIns(4,5)P_2 and PtdIns(3,4,5)P_3, at the D-5 position, with marked preference for PtdIns(4,5)P_2.[28] We therefore performed in vitro measurement of catalytic activity using full-length recombinant wild-type INPP5K and mutants affecting the phosphatase domain (p.Ile50Thr, p.Ser294del, p.Tyr300Cys) together with PtdIns(4,5)P_2diC8 as a substrate. We found that the disease-related phosphatase domain mutants and an artificial catalytically dead mutant p.Asp310Gly displayed reduced enzymatic activity (Figure 3B). While p.Tyr300Cys showed strongest impairment with activity almost as low as the catalytically dead control, the two other mutants had retained a variable degree of residual catalytic activity. Modeling of INPP5K mutants on the crystal structure of the OCRL catalytic domain showed that the mutations did not directly alter the predicted INPP5K catalytic site (Figure 3C). Instead, they affected residues that were in the folding core of the predicted crystal structure and may therefore destabilize the overall shape of the phosphatase domain, indirectly rendering the enzyme inactive.

Through metabolizing PtdIns(3,4,5)P_3, INPP5K is thought to interfere with recruitment and activation of effector proteins such as the serine/threonine kinases Akt that regulate

Muscle Weakness/ Atrophy	Best Motor Ability	CK (× Upper Normal Limit)	EMG	Muscle Biopsy	Intellectual Disability	Brain Abnormality	Respiratory/ Cardiac Involvement	Other Findings
+/NA, P > D, LL > UL	2	×4.5	ND	dystrophic	mild	–	–/–	contractures (ankle), scoliosis
+/NA, P > D, LL > UL	2	×8.5	ND	ND	moderate	ND	+/–	contractures (ankle), scoliosis
+/–, P > D, LL > UL	2	×5	ND	myopathic	moderate	–	+/–	*S. aureus* sepsis and meningitis at age 4 m
+/NA, P > D, LL > UL	2	×4.5	ND	ND	mild	–	+/–	–
+/–, P > D, LL > UL	4	×4	ND	myopathic	–	ND	+/–	spinal rigidity, contractures
+/NA, P > D, LL > UL	4	ND	ND	ND	–	ND	–/–	spinal rigidity, hyperlaxity in finger joints
+/+	2	×10	ND	myopathic	mild	–	–/–	intention tremor, microcephaly (mild)
+/+, P > D	4	×4	myopathic	dystrophic	mild	–	–/–	microcephaly, kyphosis, contractures (knee, ankle)
+/+, P > D	4	×3	ND	dystrophic	mild	–	–/–	microcephaly, kyphosis, contractures (knee, ankle), seizures
+/+, P > D, LL > UL	2	×14	myopathic	myopathic	–	–	–/–	–
+/+, P > D, LL > UL	2	ND	ND	myopathic	–	–	–/–	–
+/+, P > D	4	×12	myopathic	vacuolar myopathy	mild	mild global brain atrophy	+/–	hypogonadism

many signaling pathways.[29] INPP5K overexpression has been reported to attenuate Akt phosphorylation in response to IGF-II and insulin stimulation.[30,31] However, we noted no differences in Akt phosphorylation between IGF-II-treated human skin fibroblasts from individuals with the p.Ile50Thr mutation and control subjects (Figure S6). This discrepancy is probably related to the reported low activity of INPP5K toward PtdIns(3,4,5)P_3.[28] In line with this interpretation, we observed no INPP5K activity in the malachite green phosphatase assay with 135 μM PtdIns(3,4,5)P_3diC8 as substrate (data not shown). It seems possible that the low activity of INPP5K toward PtdIns(3,4,5)P_3 may hamper reproducible detection of downstream effects in different experiments.

Zebrafish *inpp5ka*+*inpp5kb* Knockdown Morphants Replicate Aspects of the Human Phenotype

We identified two orthologs of *INPP5K* in zebrafish, *inpp5ka* and *inpp5kb*. Treatment with specific morpholinos reduced *inpp5ka* and *inpp5kb* expression at 48 hr post fertilization (hpf) (Figure S7). Macroscopically, *inpp5ka*+*inpp5kb* MO-injected embryos displayed altered tail morphology (curled and shortened tails, Figure 4A) and severely impaired swimming and touch-evoked escape response (data not shown), whereas control MO-injected embryos and non-injected embryos were largely indistinguishable. *inpp5ka*+*inpp5kb* morphants also showed a reduction in the size of their eyes compared to non-injected and control MO-injected embryos (Figure 4B). Histologically, we observed abnormalities of skeletal muscle morphology in *inpp5ka*+*inpp5kb*-depleted embryos. Immunostaining of morphants for slow-twitch and fast-twitch fibers showed disruption of the regular chevron shape of somites, curvature and distortion of both fiber types, and abnormal myosepta (Figure 4C). In contrast, development of neuromuscular junctions progressed normally up to 48 hpf in *inpp5ka*+*inpp5kb*-deficient zebrafish embryos (Figure S8).

INPP5K Mutants Do Not Alter ER-Stress Response and BiP Interaction

We noted that there were certain similarities between *INPP5K*-associated disease and MSS, both in terms of clinical features (early-onset cataracts, skeletal muscle involvement, variable intellectual disability[2]) and muscle pathology (vacuolation and dense membranous structures

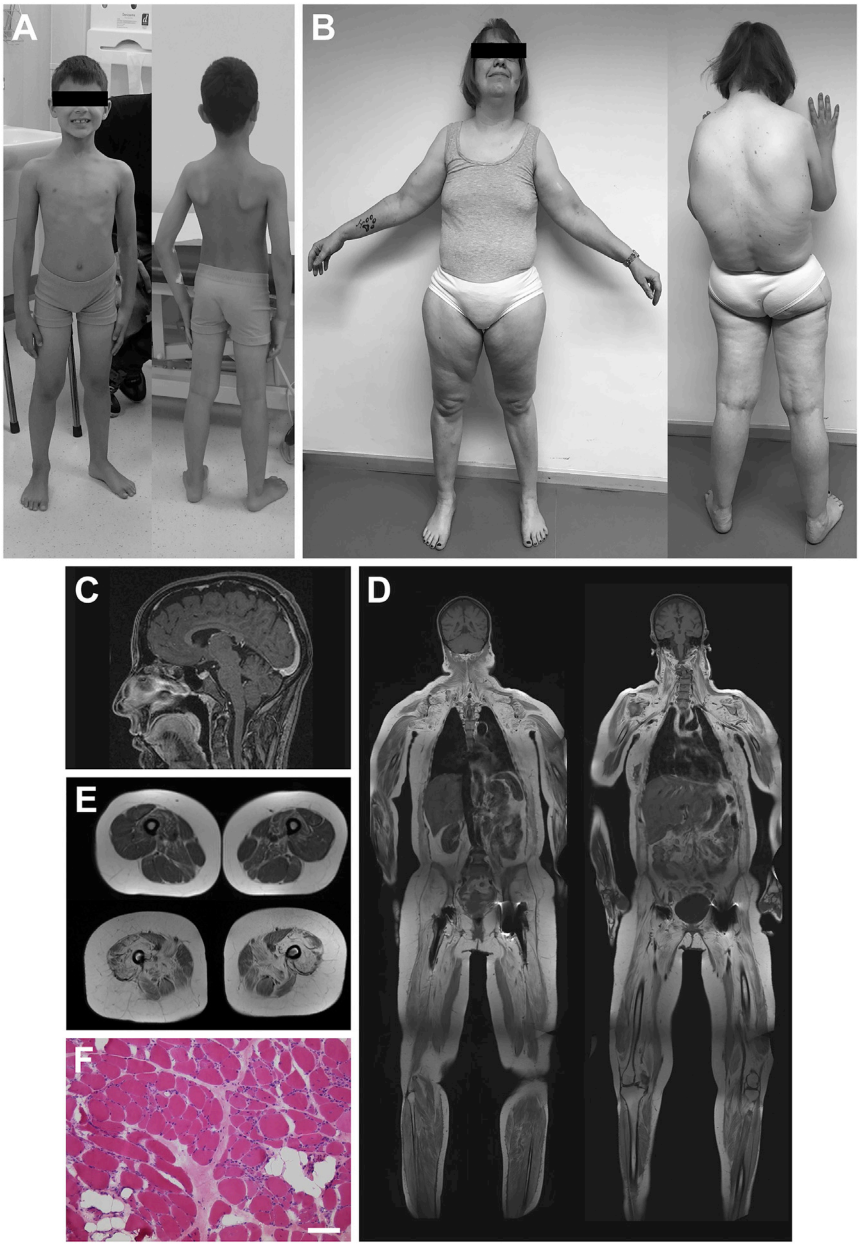

Figure 2. Clinical, Imaging, and Muscle Biopsy Findings of Individuals with Bi-allelic *INPP5K* Mutations

(A) Individual II.1, family G, at age 7 years. Note mild atrophy of the shoulder girdle muscles and posterior compartment of the thighs.

(B) Individual II.1, family H, at age 41 years. Standing independently was possible only with wide stance and the arms could not be lifted above the head (maximum arm elevation 45°) due to proximal upper and lower limb weakness. Hunchback and marked atrophy of scapuloperoneal and dorsal proximal leg muscles were noted.

(C) Individual II.1, family H, at age 25 years. Sagittal T1-weighted, contrast-enhanced cranial MRI revealed mild global brain atrophy not appropriate for age but normal cerebellar architecture.

(D) Individual II.1, family H, at age 41 years. Whole-body T1-weighted MRI showed marked muscle atrophy and fatty degeneration, predominantly in proximal arm and leg muscles.

(E) Individual II.1, family H, at age 25 years (top) and age 41 years (bottom). Cross-sectional T1-weighted MRI of the thighs revealed progressive muscle atrophy and fatty degeneration, most severe in M. vastus medialis, M. rectus femoris, M. semimembranosus, and adductor muscles.

(F) Muscle biopsy from individual II.2, family G, taken at age 3 years (site of biopsy not documented). H&E stain showed marked variation in fiber size, rounding of fibers, increased endomysial collagen, and some fatty degeneration. Scale bar represents 50 μm.

associated with nuclei[4]). Moreover, INPP5K is located at the ER[27] and MSS is related to ER dysfunction,[32,33] leading to induction of the unfolded protein response (UPR), an ER stress reaction.[34] We therefore used quantitative proteomics to measure abundances of well-validated mammalian UPR markers. However, we did not observe major differences between skin fibroblasts from individuals with bi-allelic *INPP5K* mutations and control individuals (Figure S9). Very recently, INPP5K has been reported to interact with BiP,[35] a master regulator of ER functions including the ER stress response.[36] BiP is also involved in MSS pathophysiology as its activity is controlled by the co-chaperone SIL1,[37] the protein mutant in MSS.[3,38] By coprecipitation, we confirmed binding of wild-type INPP5K to BiP, but disease-related INPP5K mutants bound equally well (Figure S10).

INPP5K Does Not Have a Major Impact on Autophagy

Some muscle biopsy findings in individuals with *INPP5K* mutations (vacuoles, sometimes rimmed, and accumulation of α-B crystallin and p62), the role of autophagy in the pathogenesis of skeletal muscular disease,[39–43] and the function of PtdIns(4,5)P_2[44] and INPP5K[45] in the regulation of autophagy prompted us to study this intracellular degradation system in a muscle biopsy (Figure S11A) and skin fibroblasts (Figures S11B and S11C) from individuals with *INPP5K* mutations. However, LC3B immunoblotting and immunofluorescence staining as well as measurement of LC3B turnover and p62 levels in fibroblasts treated with the autophagy inducer rapamycin and the lysosomal inhibitor bafilomycin A1 did not reveal specific effects of different INPP5K genotypes.

Discussion

We identified bi-allelic missense and in-frame deletion mutations in *INPP5K* in eight families with a syndrome

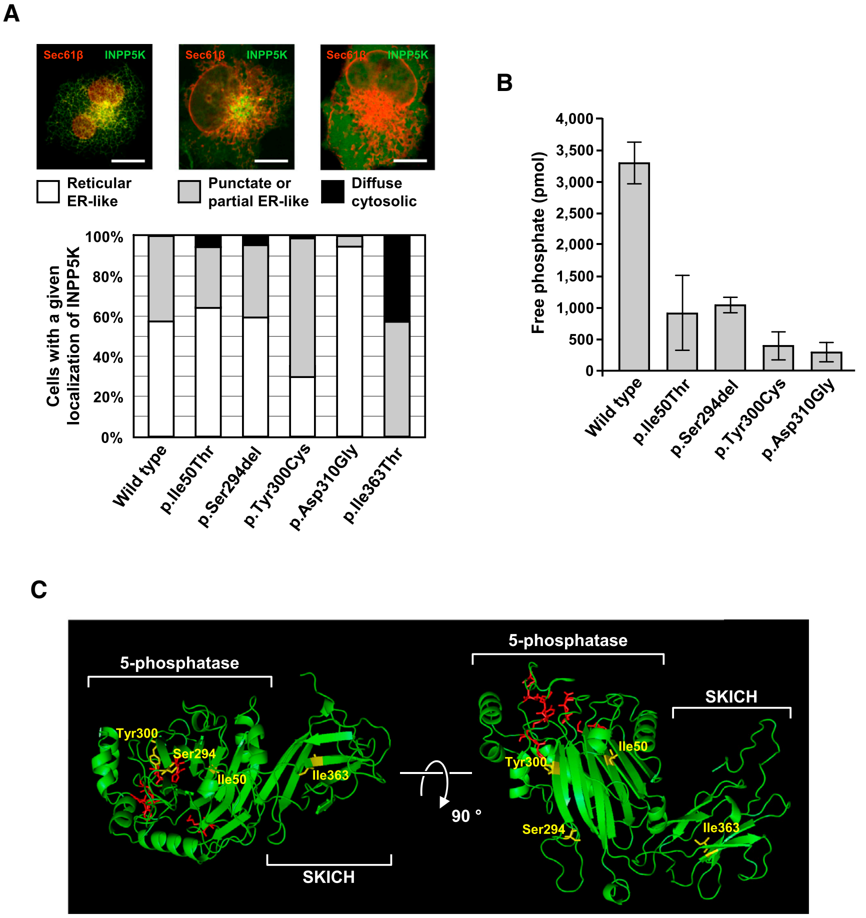

Figure 3. Structural and Functional Consequences of INPP5K Mutants

(A) Subcellular localization of INPP5K mutants. Live COS-7 cells cotransfected with the ER marker mCherry-Sec61β (red) and GFP-tagged wild-type or mutant INPP5K constructs were imaged and assigned to one of three categories. Phosphatase domain mutants did not have a strong effect on protein targeting to the ER, appearing substantially like the wild-type fusion protein. Conversely, the p.Ile363Thr mutation in the SKICH domain caused the GFP signal to become punctate or diffuse cytosolic in almost all cells. At least 60 randomly selected cells were assessed per INPP5K construct in one experiment by an observer unaware of the *INPP5K* genotype. Scale bars represent 50 μm.

(B) GST-INPP5K phosphatase activity on 135 μM PtdIns(4,5)P_2diC8. Disease mutants affecting the phosphatase domain interfere with enzyme activity. The variant p.Asp310Gly is an artificial mutant predicted to result in a catalytically dead protein through altering the active center. Bars represent mean values of three independent experiments and error bars represent standard deviations.

(C) Model of predicted INPP5K crystal structure (Phyre2). Mutated residues (yellow) were located outside the active center of the enzyme (red) but appeared to affect residues in the folding core of the 5-phosphatase domain and in the SKICH motif.

consisting of CMD, early-onset cataracts, and mild intellectual disability. The pathogenic relevance of identified *INPP5K* variants is supported by segregation with disease in several families, restriction to case subjects or extremely low prevalence in control subjects, impaired phosphatase activity for PtdIns(4,5)P_2, and aberrant subcellular localization of mutant protein. The absence of *INPP5K* truncation mutations with clear loss of function may imply that variants in our series represent hypomorphic alleles; this is further supported by embryonic lethality in homozygous constitutive *Inpp5k* knockout mice.[46] Replication of the human phenotype in zebrafish *inpp5ka*+*inpp5kb* double morphants, which manifested skeletal muscle and ocular abnormalities, lends further credence to this concept, as targeting gene expression by morpholinos usually does not result in complete deficiency of the respective proteins.

The clinical presentation of individuals with bi-allelic *INPP5K* mutations was relatively homogeneous. The most salient features were bilateral cataracts that required surgery in the first years of life, predominantly proximal muscle weakness from birth, delayed motor or global development, first followed by rather stationary course of the disease but later progression to loss of ambulation, mild intellectual disability, and elevated serum CK levels. Muscle biopsies of nine affected individuals revealed largely nonspecific dystrophic features consisting of variation in fiber size, fatty replacement, and fibrosis. Clinical and muscle biopsy findings of similar cases have been reported earlier,[47,48] and in one of these families we identified *INPP5K* mutations (family F). High serum CK levels and muscle histology were consistent with CMD rather than a form of clinically similar congenital myopathy, which typically features normal or near-normal serum CK concentrations and histological evidence of developmental or structural muscle changes.[49] Muscle pathology in *inpp5ka*+*inpp5kb* zebrafish morphants was also indicative of skeletal muscle degeneration, again supporting a muscular dystrophy in individuals with *INPP5K* mutations and reinforcing the view that zebrafish can be used to model neuromuscular conditions.[50]

Although individuals with *INPP5K* mutations did not have cerebellar atrophy and ataxia, there was a clear phenotypic overlap with MSS (early cataracts, myopathy, cerebellar atrophy, variable intellectual disability). Histological examination of three diagnostic muscle biopsies from individuals with *INPP5K* mutations showed vacuoles in several myofibers, and electron microscopy of one biopsy revealed dense membranous structures associated with some myonuclei. Similar abnormalities have been reported in MSS caused by *SIL1* mutations[2–4] and *Sil1*-deficient mice.[32] Since MSS is a disease of protein processing

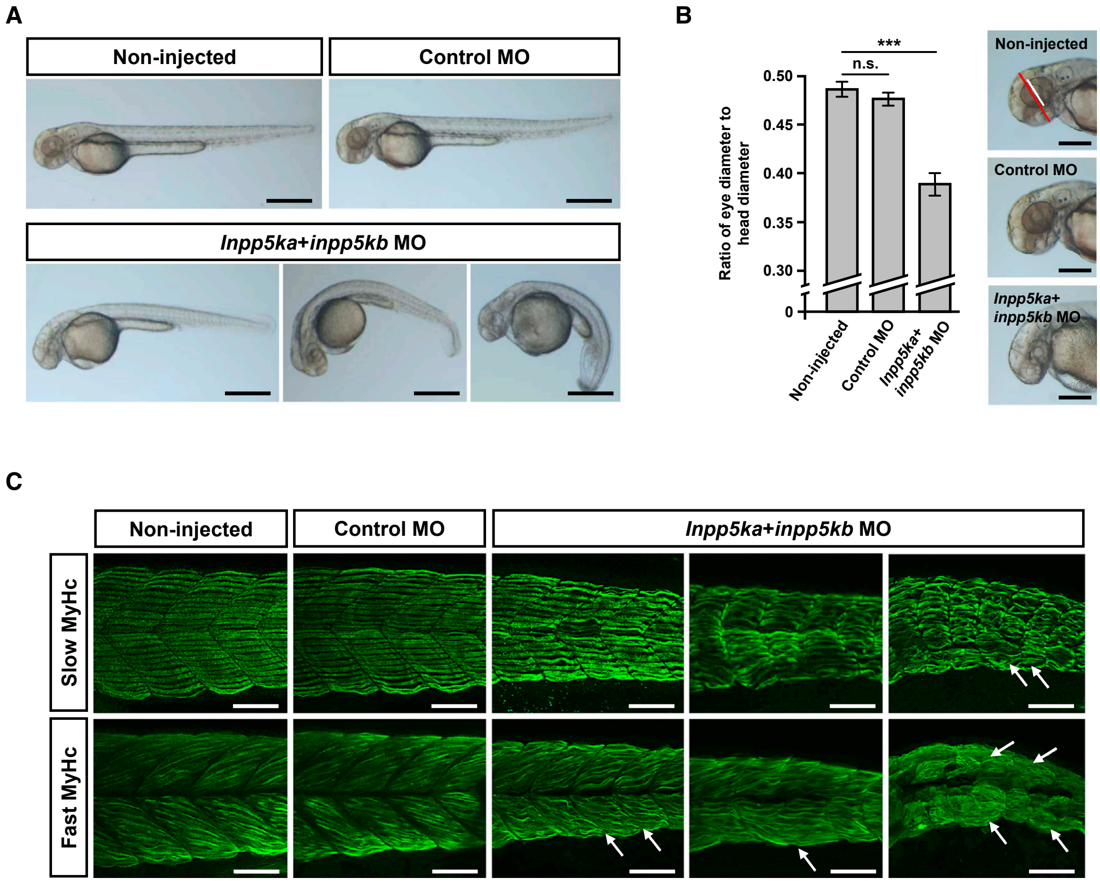

Figure 4. Defects in Zebrafish *inpp5ka+inpp5kb* Double Morphants at 48 hpf
(A) Live embryos injected with control MO, *inpp5ka* MO, and *inpp5kb* MO or left untreated (non-injected). More than 95% of living non-injected embryos and embryos injected with control MO showed no macroscopic abnormalities. Images for *inpp5ka+inpp5kb* morphants represent mild (left, 15% of living embryos), moderate (middle, 24% of living embryos), and severe (right, 44% of living embryos) defects in terms of length and curvature of tails. At least 150 living embryos were counted per condition. Scale bars represent 500 μm.
(B) Eye-to-head ratio of zebrafish embryos at 48 hpf. Eye diameter (white line) and head diameter (red line) were measured in the dorsal-ventral axis and ratios were calculated. Scale bars represent 250 μm. Graphs represent mean values of ratios obtained with ten embryos and error bars represent standard deviations. The statistical difference between non-injected and control MO-injected embryos and *inpp5ka+inpp5kb* double morphants is indicated: n.s. indicates not significant, $^{***}p < 0.001$ (one-tailed Student's t test).
(C) Whole-mount immunostainings of zebrafish embryos at 48 hpf using antibodies against slow muscle myosin heavy chain (slow MyHc) and fast muscle myosin heavy chain (fast MyHc). Images for *inpp5ka+inpp5kb* morphants represent mild, moderate, and severe phenotypes (from left to right) as staged macroscopically. *inpp5ka+inpp5kb*-deficient embryos displayed muscle fiber defects and loss of the chevron shape of somites. The normal straight alignment of slow twitch muscle fibers was disrupted in *inpp5ka+inpp5kb* double knockdown morphants. Fibers appeared wavy and deformation of myosepta was observed in severe phenotypes (arrows). Staining for fast twitch muscle fibers also showed distortion and defects of fibers and severe alterations of myosepta (arrows). Scale bars represent 50 μm.

in the ER and since INPP5K is associated with ER membranes and interacts with BiP,[35] a master regulator of ER functions,[36] we considered a pathophysiological commonality of INPP5K-related disease with MSS. However, we found no evidence for induction of the ER stress response in skin fibroblasts from individuals with bi-allelic *INPP5K* mutations and disease-causing INPP5K mutants did not interfere with interaction with BiP, suggesting still other mechanisms resulting in INPP5K-related disease.

Currently established disease mechanisms in CMD include disturbed formation of the extracellular matrix, impaired protein glycosylation, and defective phosphatidylcholine biosynthesis, as well as abnormalities of proteins of the ER and nuclear envelope.[5] Abnormalities of phosphoinositide metabolism have not yet been described in CMD but have been observed in several other neuromuscular conditions including non-dystrophic myotubular myopathy caused by mutations in *MTM1*, encoding the phosphoinositide 3-phosphatase myotubularin.[17] Phosphoinositide 5-phosphatases have not yet been implicated in skeletal muscle disorders, but have been in a number of other conditions. One example is Lowe syndrome, an X-linked disorder caused by *OCRL* mutations.[15] Boys with Lowe syndrome present with intellectual disability, epileptic seizures, kidney problems, and congenital cataracts. While cataracts and intellectual disability are seen in both Lowe syndrome and individuals with *INPP5K* mutations, there are no abnormalities of the skeletal muscle in

Lowe syndrome and individuals with *INPP5K* mutations did not have kidney disease despite the strong expression of *INPP5K* in this organ.[26]

Our data from phosphatase assays were in accordance with earlier reports that INPP5K catalyzes the removal of the 5-phosphate from PtdIns(4,5)P_2.[28] All three disease-related INPP5K mutants affecting the catalytic domain showed significantly decreased phosphoinositide phosphatase activity. Modeling of mutants on the known crystal structure of the OCRL catalytic domain suggested that they do not directly alter the catalytic site of the enzyme. Instead, they are likely to change global folding of the phosphatase domain, rendering it disordered and non-functional. The p.Ile363Thr mutant, which did not affect the phosphatase domain, showed abnormal localization away from the ER where wild-type INPP5K and the remaining mutants were located. This result is in line with this particular mutation occurring in the C-terminal SKICH domain, which is known to control INPP5K subcellular localization[27] most likely through binding to partner proteins.

Impaired enzymatic function of INPP5K mutants suggested excess PtdIns(4,5)P_2 in affected individuals' cells. PtdIns(4,5)P_2 has been linked to a wide array of molecular and cellular functions. As the substrate for receptor-regulated phospholipase C-mediated hydrolysis, its cleavage generates the secondary messengers Ins(1,4,5)P_3 and diacylglycerol, which mobilize intracellular calcium and activate protein kinase C.[7] In growth factor-activated cells, phosphoinositide 3-kinase converts PtdIns(4,5)P_2 into PtdIns(3,4,5)P_3, which is involved in the regulation of cell death, cell cycle, actin polymerization, membrane ruffling, cell migration, and secretion.[10,51–53] As an intact phospholipid, PtdIns(4,5)P_2 itself regulates membrane trafficking by recruiting protein complexes to the plasma membrane and multiple intracellular compartments.[54,55] PtdIns(4,5)P_2 functions extend to actin polymerization and focal adhesion assembly,[56] channel and transporter regulation,[57,58] virus budding,[59] exocytosis,[60] phagocytosis,[61] endocytosis,[9,60] and endosomal recycling[62] as well as endolysosomal trafficking and autophagosomal pathways.[44,63]

The role of PtdIns(4,5)P_2 in autophagy was notable as INPP5K has been linked to the control of autophagy in *Drosophila*[45] and some aspects in muscle biopsies of individuals with *INPP5K* mutations were consistent with autophagic degeneration. However, we could not corroborate these observations by studying LC3B turnover and p62 levels in a muscle biopsy and skin fibroblasts from individuals with *INPP5K* mutations. While further mechanisms related to PtdIns(4,5)P_2 will soon be tested for their relevance in INPP5K pathophysiology, it is also possible that impaired dephosphorylation of PtdIns(4,5)P_2 is not the (most) relevant aspect leading to disease, and other INPP5K substrates or unknown functions might be more important. Although we did not find differential regulation of Akt phosphorylation in IGF-II-stimulated cells from individuals with *INPP5K* mutations, INPP5K appears to be involved in PtdIns(3,4,5)P_3-dependent regulation of insulin signaling and glucose homeostasis in skeletal muscle.[64] A recent study suggested that INPP5K regulates myoblast differentiation through the IGF-II-PI 3-kinase-Akt-mTOR pathway.[30] There are discrepancies concerning the effect of impaired INPP5K activity, leading to improved myoblast differentiation,[30] contrasting with muscular dystrophy in individuals with *INPP5K* mutations. However, although unexpected in an autosomal-recessive condition, the mutant INPP5K species may have acquired characteristics of a gain of abnormal function. Despite these limitations of our study, leaving the precise pathogenic mechanisms to be determined, we have established compelling genetic evidence that INPP5K is essential for skeletal muscle structure and function.

Supplemental Data

Supplemental Data include 11 figures and 1 table and can be found with this article online at http://dx.doi.org/10.1016/j.ajhg.2017.01.024.

Acknowledgments

We wish to thank the families and study individuals for their contribution. We are grateful to Professor Heinrich Leonhardt and Dr. Joel Ryan for critical reading of the manuscript and their valuable comments. This work was supported by the Friedrich-Baur-Stiftung (to J.S.), the Wellcome Trust Institutional Strategic Support Fund (105616/Z/14/Z to L.E.S.), the Medical Research Council (MRC/N010035/1 to L.E.S.), the National Institute for Health Research Biomedical Research Centre at Great Ormond Street Hospital for Children NHS Foundation Trust and University College London (to F.M.), and the European Union Seventh Framework Programme (grant agreements 305444 [RD-Connect] and 305121 [NeurOmics] to H.L., F.M., and V.S.). The support of the Muscular Dystrophy UK to the Dubowitz Neuromuscular Centre (grant 512315 and centre grant) and of the Medical Research Council to the Neuromuscular Centres in London (UCL) and Newcastle for the Biobank is also gratefully acknowledged. Diagnostic facilities in Newcastle and London are supported by the Nationally Commissioned Highly Specialised Service (HSS) for Neuromuscular Diseases (NHS England).

B.S. has served on scientific advisory boards for Sanofi Genzyme, BioMarin, Amicus Therapeutics, and Audentes Therapeutics and receives research support from Sanofi Genzyme. C.S. is a member of the scientific advisory board of Audentes Therapeutics. F.M. has served on scientific advisory boards for Acceleron Pharma, Sanofi Genzyme, AVI BioPharma, Debiopharma Group, GlaxoSmithKline, Prosensa, Servier, Summit, and Santhera Pharmaceutical, receives research support from Trophos and GlaxoSmithKline, and has received funding for clinical trials from AVI BioPharma, PTC, and Sarepta Therapeutics.

Received: October 17, 2016
Accepted: January 12, 2017
Published: February 9, 2017

Web Resources

dbSNP (build 135 and 146), http://www.ncbi.nlm.nih.gov/projects/SNP/

ExAC Browser, http://exac.broadinstitute.org/
OMIM, http://www.omim.org/
Primer3, http://bioinfo.ut.ee/primer3
RCSB Protein Data Bank, http://www.wwpdb.org
GenBank, http://www.ncbi.nlm.nih.gov/genbank
UCSC Genome Browser, http://genome.ucsc.edu
UniProt, http://www.uniprot.org/
Zebrafish Genome Project, http://www.sanger.ac.uk/science/data/zebrafish-genome-project

References

1. Wang, C.H., Bonnemann, C.G., Rutkowski, A., Sejersen, T., Bellini, J., Battista, V., Florence, J.M., Schara, U., Schuler, P.M., Wahbi, K., et al.; International Standard of Care Committee for Congenital Muscular Dystrophy (2010). Consensus statement on standard of care for congenital muscular dystrophies. J. Child Neurol. *25*, 1559–1581.
2. Krieger, M., Roos, A., Stendel, C., Claeys, K.G., Sonmez, F.M., Baudis, M., Bauer, P., Bornemann, A., de Goede, C., Dufke, A., et al. (2013). SIL1 mutations and clinical spectrum in patients with Marinesco-Sjogren syndrome. Brain *136*, 3634–3644.
3. Senderek, J., Krieger, M., Stendel, C., Bergmann, C., Moser, M., Breitbach-Faller, N., Rudnik-Schöneborn, S., Blaschek, A., Wolf, N.I., Harting, I., et al. (2005). Mutations in SIL1 cause Marinesco-Sjögren syndrome, a cerebellar ataxia with cataract and myopathy. Nat. Genet. *37*, 1312–1314.
4. Sewry, C.A., Voit, T., and Dubowitz, V. (1988). Myopathy with unique ultrastructural feature in Marinesco-Sjögren syndrome. Ann. Neurol. *24*, 576–580.
5. Mercuri, E., and Muntoni, F. (2012). The ever-expanding spectrum of congenital muscular dystrophies. Ann. Neurol. *72*, 9–17.
6. O'Grady, G.L., Lek, M., Lamande, S.R., Waddell, L., Oates, E.C., Punetha, J., Ghaoui, R., Sandaradura, S.A., Best, H., Kaur, S., et al. (2016). Diagnosis and etiology of congenital muscular dystrophy: We are halfway there. Ann. Neurol. *80*, 101–111.
7. Berridge, M.J., and Irvine, R.F. (1989). Inositol phosphates and cell signalling. Nature *341*, 197–205.
8. De Matteis, M.A., and Godi, A. (2004). PI-loting membrane traffic. Nat. Cell Biol. *6*, 487–492.
9. Di Paolo, G., and De Camilli, P. (2006). Phosphoinositides in cell regulation and membrane dynamics. Nature *443*, 651–657.
10. Martin, T.F. (1998). Phosphoinositide lipids as signaling molecules: common themes for signal transduction, cytoskeletal regulation, and membrane trafficking. Annu. Rev. Cell Dev. Biol. *14*, 231–264.
11. Odorizzi, G., Babst, M., and Emr, S.D. (2000). Phosphoinositide signaling and the regulation of membrane trafficking in yeast. Trends Biochem. Sci. *25*, 229–235.
12. Behnia, R., and Munro, S. (2005). Organelle identity and the signposts for membrane traffic. Nature *438*, 597–604.
13. Dyson, J.M., Fedele, C.G., Davies, E.M., Becanovic, J., and Mitchell, C.A. (2012). Phosphoinositide phosphatases: just as important as the kinases. Subcell. Biochem. *58*, 215–279.
14. McCrea, H.J., and De Camilli, P. (2009). Mutations in phosphoinositide metabolizing enzymes and human disease. Physiology (Bethesda) *24*, 8–16.
15. Attree, O., Olivos, I.M., Okabe, I., Bailey, L.C., Nelson, D.L., Lewis, R.A., McInnes, R.R., and Nussbaum, R.L. (1992). The Lowe's oculocerebrorenal syndrome gene encodes a protein highly homologous to inositol polyphosphate-5-phosphatase. Nature *358*, 239–242.
16. Narkis, G., Ofir, R., Landau, D., Manor, E., Volokita, M., Hershkowitz, R., Elbedour, K., and Birk, O.S. (2007). Lethal contractural syndrome type 3 (LCCS3) is caused by a mutation in PIP5K1C, which encodes PIPKI gamma of the phophatidylinsitol pathway. Am. J. Hum. Genet. *81*, 530–539.
17. Laporte, J., Hu, L.J., Kretz, C., Mandel, J.L., Kioschis, P., Coy, J.F., Klauck, S.M., Poustka, A., and Dahl, N. (1996). A gene mutated in X-linked myotubular myopathy defines a new putative tyrosine phosphatase family conserved in yeast. Nat. Genet. *13*, 175–182.
18. Bolino, A., Muglia, M., Conforti, F.L., LeGuern, E., Salih, M.A., Georgiou, D.M., Christodoulou, K., Hausmanowa-Petrusewicz, I., Mandich, P., Schenone, A., et al. (2000). Charcot-Marie-Tooth type 4B is caused by mutations in the gene encoding myotubularin-related protein-2. Nat. Genet. *25*, 17–19.
19. Azzedine, H., Bolino, A., Taïeb, T., Birouk, N., Di Duca, M., Bouhouche, A., Benamou, S., Mrabet, A., Hammadouche, T., Chkili, T., et al. (2003). Mutations in MTMR13, a new pseudophosphatase homologue of MTMR2 and Sbf1, in two families with an autosomal recessive demyelinating form of Charcot-Marie-Tooth disease associated with early-onset glaucoma. Am. J. Hum. Genet. *72*, 1141–1153.
20. Senderek, J., Bergmann, C., Weber, S., Ketelsen, U.P., Schorle, H., Rudnik-Schöneborn, S., Büttner, R., Buchheim, E., and Zerres, K. (2003). Mutation of the SBF2 gene, encoding a novel member of the myotubularin family, in Charcot-Marie-Tooth neuropathy type 4B2/11p15. Hum. Mol. Genet. *12*, 349–356.
21. Chow, C.Y., Zhang, Y., Dowling, J.J., Jin, N., Adamska, M., Shiga, K., Szigeti, K., Shy, M.E., Li, J., Zhang, X., et al. (2007). Mutation of FIG4 causes neurodegeneration in the pale tremor mouse and patients with CMT4J. Nature *448*, 68–72.
22. Ho, S.N., Hunt, H.D., Horton, R.M., Pullen, J.K., and Pease, L.R. (1989). Site-directed mutagenesis by overlap extension using the polymerase chain reaction. Gene *77*, 51–59.
23. Zurek, N., Sparks, L., and Voeltz, G. (2011). Reticulon short hairpin transmembrane domains are used to shape ER tubules. Traffic *12*, 28–41.
24. Kelley, L.A., Mezulis, S., Yates, C.M., Wass, M.N., and Sternberg, M.J. (2015). The Phyre2 web portal for protein modeling, prediction and analysis. Nat. Protoc. *10*, 845–858.
25. Kimmel, C.B., Ballard, W.W., Kimmel, S.R., Ullmann, B., and Schilling, T.F. (1995). Stages of embryonic development of the zebrafish. Dev. Dyn. *203*, 253–310.
26. Ijuin, T., Mochizuki, Y., Fukami, K., Funaki, M., Asano, T., and Takenawa, T. (2000). Identification and characterization of a novel inositol polyphosphate 5-phosphatase. J. Biol. Chem. *275*, 10870–10875.
27. Gurung, R., Tan, A., Ooms, L.M., McGrath, M.J., Huysmans, R.D., Munday, A.D., Prescott, M., Whisstock, J.C., and Mitchell, C.A. (2003). Identification of a novel domain in two mammalian inositol-polyphosphate 5-phosphatases that mediates membrane ruffle localization. The inositol 5-phosphatase skip localizes to the endoplasmic reticulum and translocates to membrane ruffles following epidermal growth factor stimulation. J. Biol. Chem. *278*, 11376–11385.
28. Schmid, A.C., Wise, H.M., Mitchell, C.A., Nussbaum, R., and Woscholski, R. (2004). Type II phosphoinositide 5-phosphatases have unique sensitivities towards fatty acid composition and head group phosphorylation. FEBS Lett. *576*, 9–13.

29. Stokoe, D., Stephens, L.R., Copeland, T., Gaffney, P.R., Reese, C.B., Painter, G.F., Holmes, A.B., McCormick, F., and Hawkins, P.T. (1997). Dual role of phosphatidylinositol-3,4,5-trisphosphate in the activation of protein kinase B. Science *277*, 567–570.
30. Ijuin, T., and Takenawa, T. (2012). Role of phosphatidylinositol 3,4,5-trisphosphate (PIP3) 5-phosphatase skeletal muscle- and kidney-enriched inositol polyphosphate phosphatase (SKIP) in myoblast differentiation. J. Biol. Chem. *287*, 31330–31341.
31. Ijuin, T., and Takenawa, T. (2003). SKIP negatively regulates insulin-induced GLUT4 translocation and membrane ruffle formation. Mol. Cell. Biol. *23*, 1209–1220.
32. Roos, A., Buchkremer, S., Kollipara, L., Labisch, T., Gatz, C., Zitzelsberger, M., Brauers, E., Nolte, K., Schröder, J.M., Kirschner, J., et al. (2014). Myopathy in Marinesco-Sjögren syndrome links endoplasmic reticulum chaperone dysfunction to nuclear envelope pathology. Acta Neuropathol. *127*, 761–777.
33. Zhao, L., Rosales, C., Seburn, K., Ron, D., and Ackerman, S.L. (2010). Alteration of the unfolded protein response modifies neurodegeneration in a mouse model of Marinesco-Sjögren syndrome. Hum. Mol. Genet. *19*, 25–35.
34. Ma, Y., and Hendershot, L.M. (2001). The unfolding tale of the unfolded protein response. Cell *107*, 827–830.
35. Ijuin, T., Hatano, N., and Takenawa, T. (2016). Glucose-regulated protein 78 (GRP78) binds directly to PIP3 phosphatase SKIP and determines its localization. Genes Cells *21*, 457–465.
36. Hendershot, L.M. (2004). The ER function BiP is a master regulator of ER function. Mt. Sinai J. Med. *71*, 289–297.
37. Chung, K.T., Shen, Y., and Hendershot, L.M. (2002). BAP, a mammalian BiP-associated protein, is a nucleotide exchange factor that regulates the ATPase activity of BiP. J. Biol. Chem. *277*, 47557–47563.
38. Anttonen, A.K., Mahjneh, I., Hämäläinen, R.H., Lagier-Tourenne, C., Kopra, O., Waris, L., Anttonen, M., Joensuu, T., Kalimo, H., Paetau, A., et al. (2005). The gene disrupted in Marinesco-Sjögren syndrome encodes SIL1, an HSPA5 cochaperone. Nat. Genet. *37*, 1309–1311.
39. Cullup, T., Kho, A.L., Dionisi-Vici, C., Brandmeier, B., Smith, F., Urry, Z., Simpson, M.A., Yau, S., Bertini, E., McClelland, V., et al. (2013). Recessive mutations in EPG5 cause Vici syndrome, a multisystem disorder with defective autophagy. Nat. Genet. *45*, 83–87.
40. Grumati, P., Coletto, L., Sabatelli, P., Cescon, M., Angelin, A., Bertaggia, E., Blaauw, B., Urciuolo, A., Tiepolo, T., Merlini, L., et al. (2010). Autophagy is defective in collagen VI muscular dystrophies, and its reactivation rescues myofiber degeneration. Nat. Med. *16*, 1313–1320.
41. Nishino, I., Fu, J., Tanji, K., Yamada, T., Shimojo, S., Koori, T., Mora, M., Riggs, J.E., Oh, S.J., Koga, Y., et al. (2000). Primary LAMP-2 deficiency causes X-linked vacuolar cardiomyopathy and myopathy (Danon disease). Nature *406*, 906–910.
42. Ramachandran, N., Munteanu, I., Wang, P., Ruggieri, A., Rilstone, J.J., Israelian, N., Naranian, T., Paroutis, P., Guo, R., Ren, Z.P., et al. (2013). VMA21 deficiency prevents vacuolar ATPase assembly and causes autophagic vacuolar myopathy. Acta Neuropathol. *125*, 439–457.
43. De Palma, C., Morisi, F., Cheli, S., Pambianco, S., Cappello, V., Vezzoli, M., Rovere-Querini, P., Moggio, M., Ripolone, M., Francolini, M., et al. (2014). Autophagy as a new therapeutic target in Duchenne muscular dystrophy. Cell Death Dis. *5*, e1363.
44. Moreau, K., Ravikumar, B., Puri, C., and Rubinsztein, D.C. (2012). Arf6 promotes autophagosome formation via effects on phosphatidylinositol 4,5-bisphosphate and phospholipase D. J. Cell Biol. *196*, 483–496.
45. Zirin, J., Nieuwenhuis, J., Samsonova, A., Tao, R., and Perrimon, N. (2015). Regulators of autophagosome formation in *Drosophila* muscles. PLoS Genet. *11*, e1005006.
46. Ijuin, T., Yu, Y.E., Mizutani, K., Pao, A., Tateya, S., Tamori, Y., Bradley, A., and Takenawa, T. (2008). Increased insulin action in SKIP heterozygous knockout mice. Mol. Cell. Biol. *28*, 5184–5195.
47. Reed, U.C., Tsanaclis, A.M., Vainzof, M., Marie, S.K., Carvalho, M.S., Roizenblatt, J., Pedreira, C.C., Diament, A., and Levy, J.A. (1999). Merosin-positive congenital muscular dystrophy in two siblings with cataract and slight mental retardation. Brain Dev. *21*, 274–278.
48. Topaloğlu, H., Yetük, M., Talim, B., Akçören, Z., and Cağlar, M. (1997). Merosin-positive congenital muscular dystrophy with mental retardation and cataracts: a new entity in two families. Eur. J. Paediatr. Neurol. *1*, 127–131.
49. North, K.N., Wang, C.H., Clarke, N., Jungbluth, H., Vainzof, M., Dowling, J.J., Amburgey, K., Quijano-Roy, S., Beggs, A.H., Sewry, C., et al.; International Standard of Care Committee for Congenital Myopathies (2014). Approach to the diagnosis of congenital myopathies. Neuromuscul. Disord. *24*, 97–116.
50. Gibbs, E.M., Horstick, E.J., and Dowling, J.J. (2013). Swimming into prominence: the zebrafish as a valuable tool for studying human myopathies and muscular dystrophies. FEBS J. *280*, 4187–4197.
51. Simonsen, A., Wurmser, A.E., Emr, S.D., and Stenmark, H. (2001). The role of phosphoinositides in membrane transport. Curr. Opin. Cell Biol. *13*, 485–492.
52. Rameh, L.E., and Cantley, L.C. (1999). The role of phosphoinositide 3-kinase lipid products in cell function. J. Biol. Chem. *274*, 8347–8350.
53. Vanhaesebroeck, B., Leevers, S.J., Ahmadi, K., Timms, J., Katso, R., Driscoll, P.C., Woscholski, R., Parker, P.J., and Waterfield, M.D. (2001). Synthesis and function of 3-phosphorylated inositol lipids. Annu. Rev. Biochem. *70*, 535–602.
54. Lemmon, M.A. (2008). Membrane recognition by phospholipid-binding domains. Nat. Rev. Mol. Cell Biol. *9*, 99–111.
55. McLaughlin, S., Wang, J., Gambhir, A., and Murray, D. (2002). PIP(2) and proteins: interactions, organization, and information flow. Annu. Rev. Biophys. Biomol. Struct. *31*, 151–175.
56. Yin, H.L., and Janmey, P.A. (2003). Phosphoinositide regulation of the actin cytoskeleton. Annu. Rev. Physiol. *65*, 761–789.
57. Balla, T. (2009). Regulation of Ca2+ entry by inositol lipids in mammalian cells by multiple mechanisms. Cell Calcium *45*, 527–534.
58. Suh, B.C., and Hille, B. (2008). PIP2 is a necessary cofactor for ion channel function: how and why? Annu. Rev. Biophys. *37*, 175–195.
59. Saad, J.S., Miller, J., Tai, J., Kim, A., Ghanam, R.H., and Summers, M.F. (2006). Structural basis for targeting HIV-1 Gag proteins to the plasma membrane for virus assembly. Proc. Natl. Acad. Sci. USA *103*, 11364–11369.

60. Martin, T.F. (2001). PI(4,5)P(2) regulation of surface membrane traffic. Curr. Opin. Cell Biol. *13*, 493–499.
61. Grinstein, S. (2010). Imaging signal transduction during phagocytosis: phospholipids, surface charge, and electrostatic interactions. Am. J. Physiol. Cell Physiol. *299*, C876–C881.
62. Kim, S., Kim, H., Chang, B., Ahn, N., Hwang, S., Di Paolo, G., and Chang, S. (2006). Regulation of transferrin recycling kinetics by PtdIns[4,5]P2 availability. FASEB J. *20*, 2399–2401.
63. De Leo, M.G., Staiano, L., Vicinanza, M., Luciani, A., Carissimo, A., Mutarelli, M., Di Campli, A., Polishchuk, E., Di Tullio, G., Morra, V., et al. (2016). Autophagosome-lysosome fusion triggers a lysosomal response mediated by TLR9 and controlled by OCRL. Nat. Cell Biol. *18*, 839–850.
64. Ijuin, T., and Takenawa, T. (2012). Regulation of insulin signaling and glucose transporter 4 (GLUT4) exocytosis by phosphatidylinositol 3,4,5-trisphosphate (PIP3) phosphatase, skeletal muscle, and kidney enriched inositol polyphosphate phosphatase (SKIP). J. Biol. Chem. *287*, 6991–6999.

The American Journal of Human Genetics, Volume *100*

Supplemental Data

Mutations in *INPP5K*, Encoding a Phosphoinositide 5-Phosphatase, Cause Congenital Muscular Dystrophy with Cataracts and Mild Cognitive Impairment

Manuela Wiessner, Andreas Roos, Christopher J. Munn, Ranjith Viswanathan, Tamieka Whyte, Dan Cox, Benedikt Schoser, Caroline Sewry, Helen Roper, Rahul Phadke, Chiara Marini Bettolo, Rita Barresi, Richard Charlton, Carsten G. Bönnemann, Osório Abath Neto, Umbertina C. Reed, Edmar Zanoteli, Cristiane Araújo Martins Moreno, Birgit Ertl-Wagner, Rolf Stucka, Christian De Goede, Tamiris Borges da Silva, Denisa Hathazi, Margherita Dell'Aica, René P. Zahedi, Simone Thiele, Juliane Müller, Helen Kingston, Susanna Müller, Elizabeth Curtis, Maggie C. Walter, Tim M. Strom, Volker Straub, Kate Bushby, Francesco Muntoni, Laura E. Swan, Hanns Lochmüller, and Jan Senderek

Figure S1. Genome-wide linkage analysis of family A.

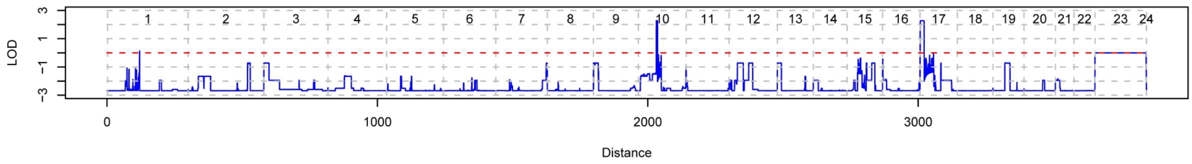

Genome-wide SNP genotyping was performed using the Illumina 300K chip (Illumina). Multipoint linkage was calculated with ALLEGRO [1] assuming autosomal recessive inheritance, a frequency of the deleterious allele of 0.001, and complete penetrance. The region of interest on chr10 spanned 7.18 Mb and contained 42 RefSeq genes. The candidate region on chr17 spanned 5.58 Mb and contained 98 RefSeq genes.

Figure S2. *INPP5K* mutations identified in individuals with CMD and cataracts.

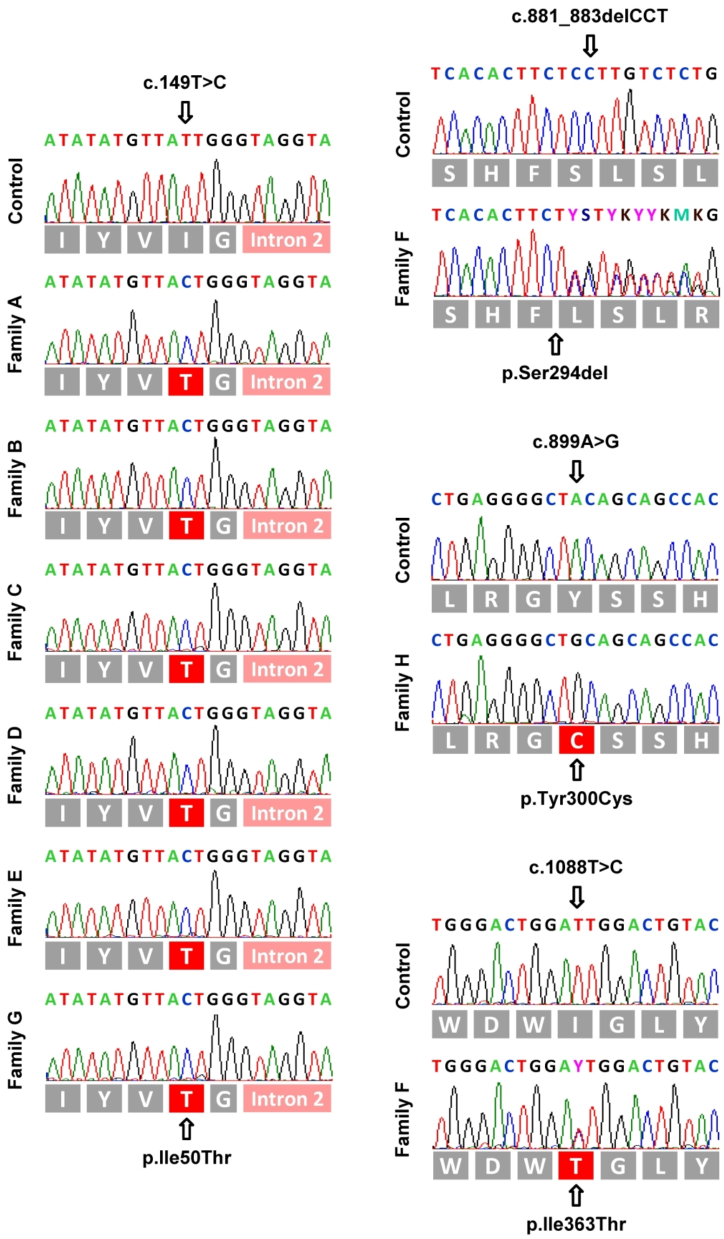

Sequencing electropherograms for comparison of nucleotide sequences of individuals affected with CMD and cataracts and unaffected control individuals.

Figure S3. Tissue distribution and localization of INPP5K.

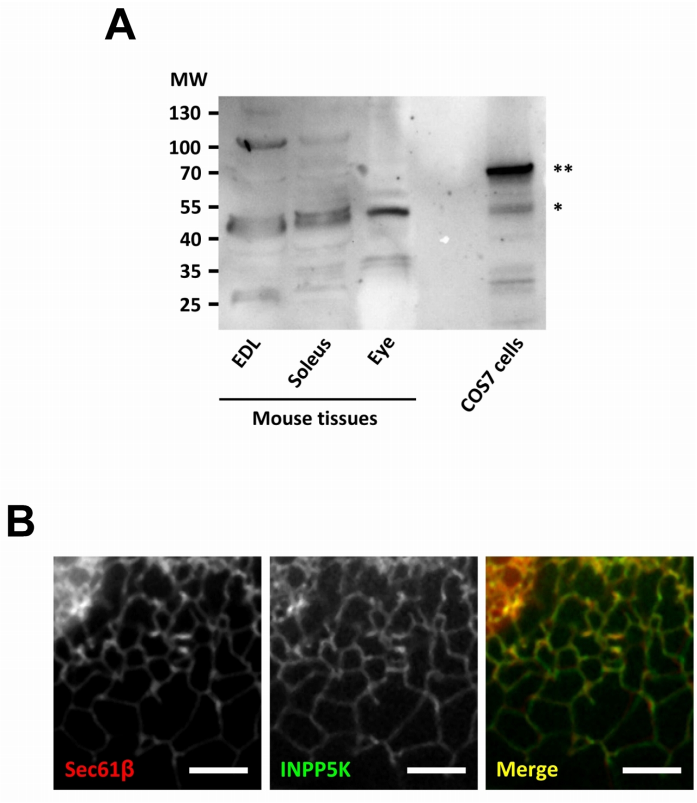

(A) Tissues from young adult C57BL/6J mice (P56) were homogenized with a rotor-stator homogenizer (TissueRuptor, Qiagen) in 1x PBS containing 1% SDS as well as protease (Complete Protease Inhibitor; Roche Diagnostics) and phosphatase inhibitors (PhosSTOP; Roche Diagnostics). Protein preparations were incubated at 95°C for 5 min, debris was removed by 5 min centrifugation at 14,000 rpm at 4°C, and protein concentrations in the supernatants were measured with a BCA protein assay (Thermo Fisher Scientific). Protein samples were separated by sodium dodecyl sulfate (SDS)-polyacrylamide gel electrophoresis and transferred to a nitrocellulose membrane (Protran; GE Healthcare). Transferred proteins were blocked for 1 hr at RT in TBS-T with 5% non-fat milk and then incubated overnight at 4°C with goat anti-INPP5K antibody (C19; Santa Cruz Biotechnology; 1:200). Subsequently, the blot was incubated with horseradish peroxidise (HRP)-conjugated donkey anti-goat IgG antibody (Jackson ImmunoResearch; 1:10,000) for 1 hr at RT. Bands were detected with an ECL Detection Kit (GE Healthcare). A lysate from COS-7 cells transfected with an expression vector coding for a GFP-INPP5K fusion protein was used as a control. EDL = extensor digitorum longus muscle; soleus = soleus muscle; eye = whole eye tissue; * = endogenous INPP5K; ** = GFP-INPP5K fusion protein; MW = Molecular weight in kilo Dalton. **(B)** In live-imaged transfected COS-7 cells, the ER marker mCherry-Sec61β (red) colocalizes extensively with wild-type GFP-INPP5K (green). Images show a higher magnification of a detail of Figure 3A (left panel). Scale bars = 10 μm.

Figure S4. Muscle biopsies from individuals with bi-allelic *INPP5K* mutations.

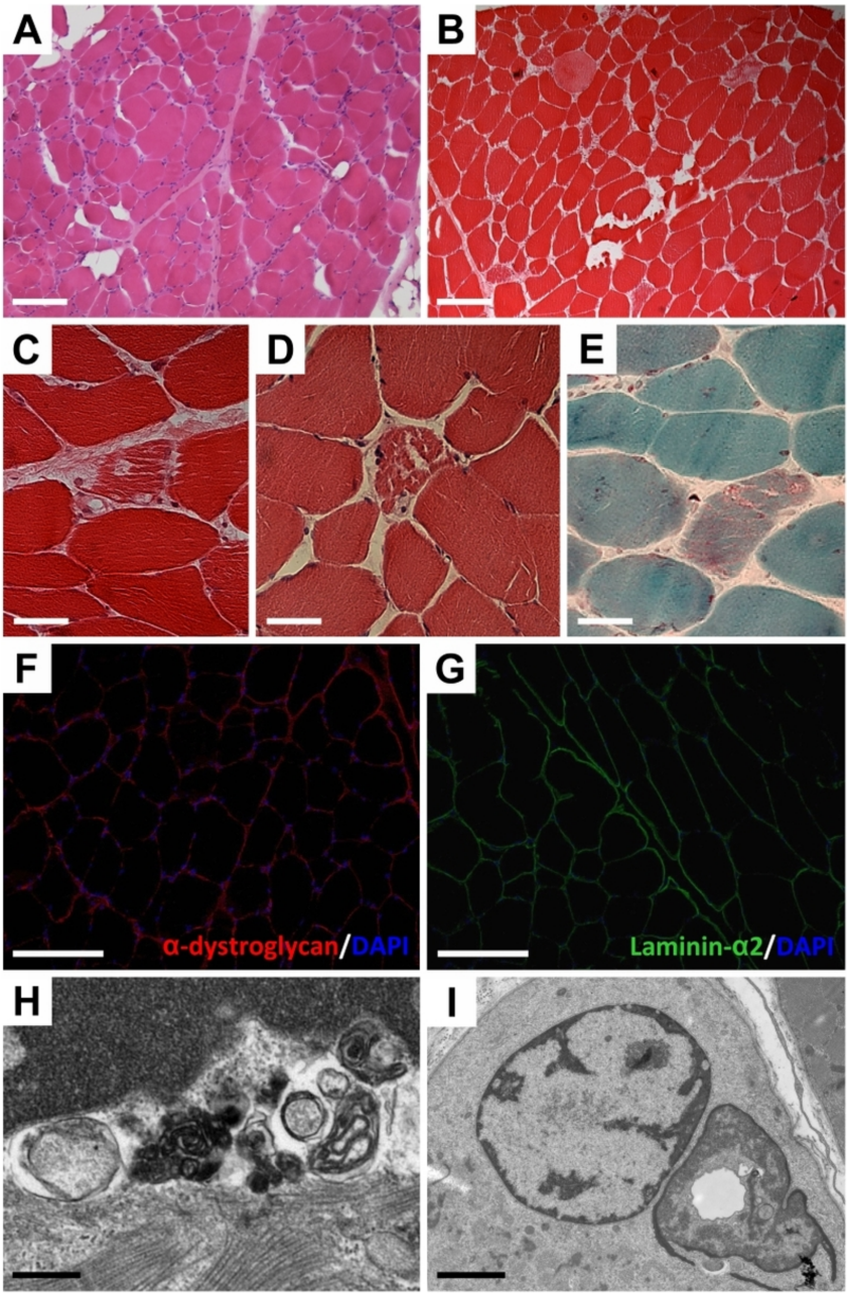

(A, B) H&E stain revealing variation in fiber size, rounding of fibers, increased endomysial collagen and some degree of fatty degeneration. **(C, D)** H&E stain, **(E)** Gomori trichrome stain showing vacuolated muscle fibers. With Gomori trichrome, some vacuoles contained red-stained material. **(F)** α-dystroglycan (clone VIA4-1) and **(G)** Laminin-α2 (clone 2H5) immunostainings gave positive results. **(H)** Electron micrograph showing a vacuole associated with myelin-like whorls and osmiophilic material. **(I)** Electron micrograph of nuclei in a degenerating muscle fiber, one with dense peripheral heterochromatin (left) and one partially surrounded by an electron-dense membranous structure (right). **(A)**: left M. vastus lateralis from individual IV.3, family A, taken at age 6 years, **(B-E, F, G)**: left M. biceps brachii from individual II.1, family H, taken at age 25 years), **(H, I)**: left quadriceps muscle from individual II.3, family B, taken at age 4 years). Scale bars **(A, B, F, G)** = 50 µm, **(C-E)** = 10 µm, **(H)** = 0.5 µm, **(I)** = 2 µm.

Figure S5. Levels of wild-type and mutant INPP5K in transfected cells.

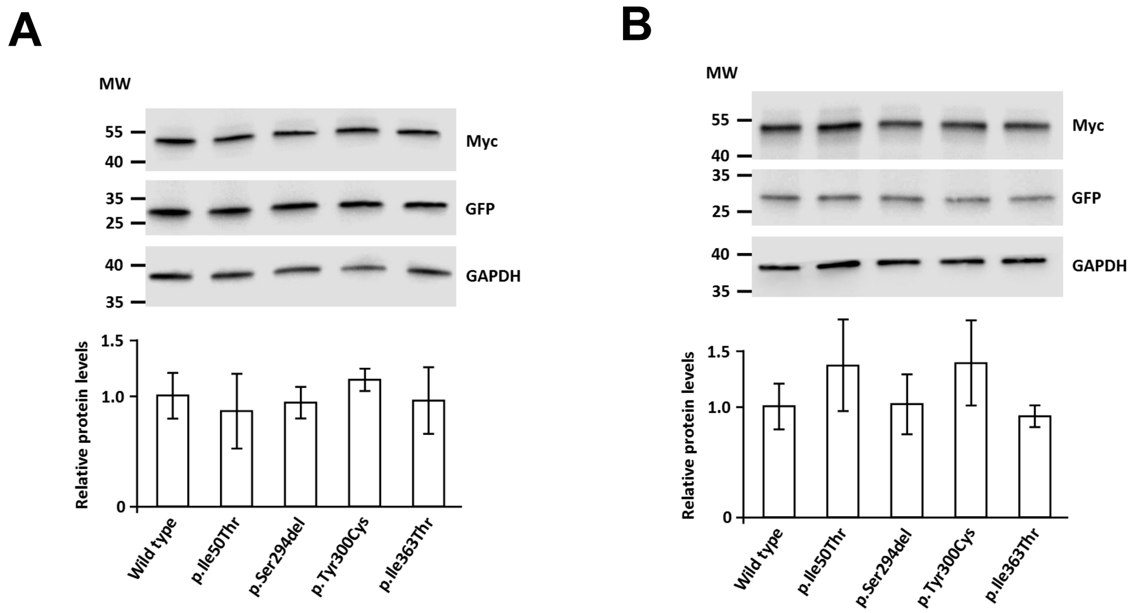

(A) Human *INPP5K* cDNA was amplified from human skeletal muscle mRNA and cloned in pCMV-Myc. INPP5K mutants were generated by site-directed mutagenesis and verified by sequencing. C2C12 cells were cultured in DMEM containing 10% fetal calf serum, 2 mM glutamine, 40 U/mL penicillin and 0.04 mg/mL streptomycin. Cells were cotransfected with Myc-tagged INPP5K expression constructs and an empty GFP expression plasmid (transfection efficiency control) using Amaxa Cell Line Nucleofector Kit V (Lonza). Cultured cells were lysed in cell lysis buffer (1% SDS, 10 mM Tris-HCl, pH 7.4) containing protease and phosphatase inhibitors. Protein preparations were incubated at 95°C for 5 min, debris was removed by 5 min centrifugation at 14,000 rpm at 4°C, and protein concentrations in the supernatants were measured with a BCA protein assay. Equal amounts of total protein were separated by SDS-polyacrylamide gel electrophoresis and transferred to nitrocellulose membranes. Membranes were blocked for 1 hr at RT in TBS-T with 5% non-fat milk and then incubated overnight at 4°C with primary antibodies mouse anti-Myc (Clontech; 1:1,000), rabbit anti-GFP (Abcam; 1:1,000) and rabbit anti-GAPDH (Merck Millipore; 1:500). Subsequently, blots were incubated with HRP-conjugated goat anti-mouse IgG and goat anti-rabbit IgG antibodies (both from Invitrogen; 1:5,000) for 1 hr at RT. Bands were detected with an ECL Detection Kit. Densitometry to quantify INPP5K levels was performed using ImageJ software. Signals obtained for INPP5K were normalized to GFP (transfection efficiency control) and GAPDH (loading control). Bars represent means of three independent experiments and error bars represent standard deviations. MW = Molecular weight in kilo Dalton. **(B)** Same analysis using COS-7 cells. Experimental conditions were identical to those described in (A) except for transfection of cells using JetPei Transfection Reagent (Peqlab) instead of nucleofection.

Figure S6. Akt phosphorylation in IGF-II-stimulated primary human skin fibroblasts.

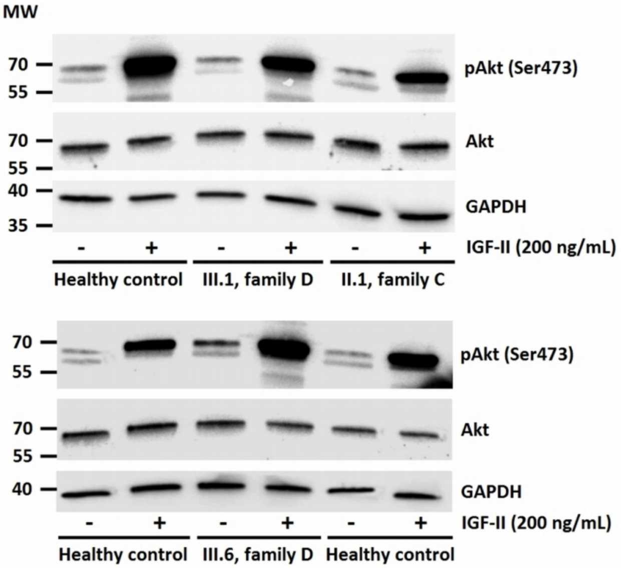

Skin fibroblasts from three individuals homozygous for the *INPP5K* mutation p.Ile50Thr (II.1, family C, III.1, family D and III.6, family D) and three healthy subjects were obtained from the MRC Centre for Neuromuscular Diseases Biobank, Newcastle, UK. Cells were cultured in DMEM containing 10% fetal calf serum, 2 mM glutamine, 40 U/mL penicillin and 0.04 mg/mL streptomycin. Prior to cell lysis, cultures were kept in serum-free DMEM for 48 hr and then stimulated with 200 ng/mL IGF-II (Merck Millipore) for 1 hr [2]. Cells were lysed in cell lysis buffer supplemented with protease and phosphatase inhibitors. Protein preparations were incubated at 95°C for 5 min, debris was removed by 5 min centrifugation at 14,000 rpm at 4°C, and protein concentrations in the supernatants were measured with a BCA protein assay. Equal amounts of total protein were run on SDS-polyacrylamide gels and blotted onto nitrocellulose membranes. Membranes were blocked for 1 hr at RT in TBS-T with 5% non-fat milk and then incubated overnight at 4°C with primary antibodies mouse anti-Akt, rabbit anti-phospho-Akt (Ser473) (both from Cell Signaling Technology; 1:2,000) and rabbit anti-GAPDH. Subsequently, blots were incubated with HRP-conjugated goat anti-mouse IgG and goat anti-rabbit IgG antibodies for 1 hr at RT. Bands were detected with an ECL Detection Kit. MW = Molecular weight in kilo Dalton.

Figure S7. MO-mediated double knockdown of *inpp5ka* and *inpp5kb* in zebrafish.

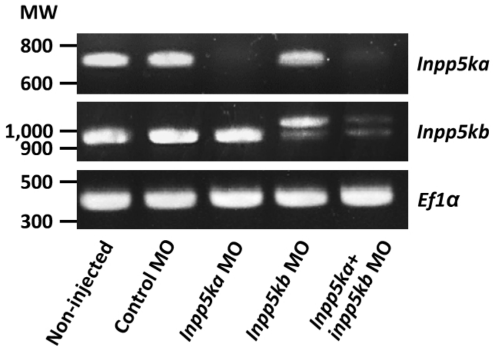

RT-PCR analysis of *inpp5ka* and *inpp5kb* transcripts from 48-hpf embryos injected with splice-blocking *inpp5ka* and *inpp5kb* MOs. RNA was isolated with Trizol reagent (Invitrogen). Reverse transcription was performed with the Superscript III First-Strand Synthesis System (Invitrogen). Target sequences were PCR-amplified using specific oligonucleotide primers, run on agarose gels and detected by SafeView Nucleic Acid Stain (NBS Biologicals). Top panel: Downregulation of *inpp5ka* in both the single and double knockdown was demonstrated with *inpp5ka*-specific PCR primers. Middle panel: Mis-spliced *inpp5kb* transcripts alongside small amount of retained wild-type transcript were detected in the single and double knockdown with *inpp5kb*-specific PCR primers. Bottom panel: *Ef1α* was used as an internal RT-PCR control to exclude variations in cDNA synthesis quality. MW = Molecular weight in base pairs.

Figure S8. Neuromuscular junction morphology in *inpp5ka+inpp5kb* morphants.

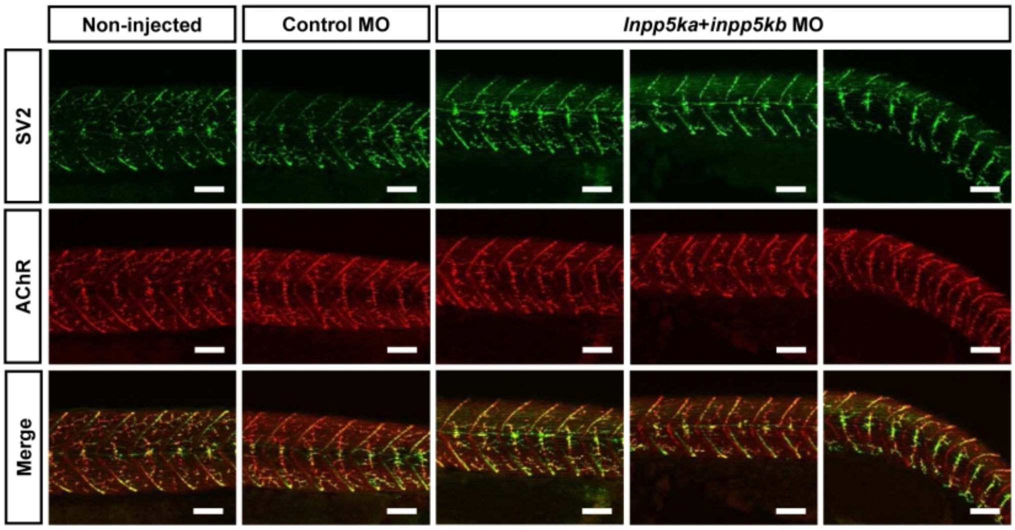

For whole-mount immunofluorescence staining, 48-hpf zebrafish embryos were fixed in 4% paraformaldehyde in PBS at 4°C overnight and blocked for 1 hr at RT in 5% horse serum in PBS, 0.1% Tween-20. Presynaptic motor nerve endings were visualized by incubation with mouse anti-synaptic vesicle protein 2 antibody (SV2, DSHB; 1:200) overnight at 4°C followed by Alexa Fluor 488-conjugated goat anti-mouse IgG antibody for 1 hr at RT. Acetylcholine receptors (AChR) were labelled with Alexa Fluor 594-conjugated α-bungarotoxin (Thermo Fisher Scientific; 1:1,000). Images were captured with a Nikon A1R confocal microscope (Nikon). Images for *inpp5ka+inpp5kb* morphants represent mild, moderate and severe phenotypes (from left to right) as staged macroscopically. Both in controls and in *inpp5ka+inpp5kb* morphants, motor axons had made branches into somites where they formed contacts with AChR clusters. Scale bars = 50 µm.

Figure S9. Quantification of ER stress response markers.

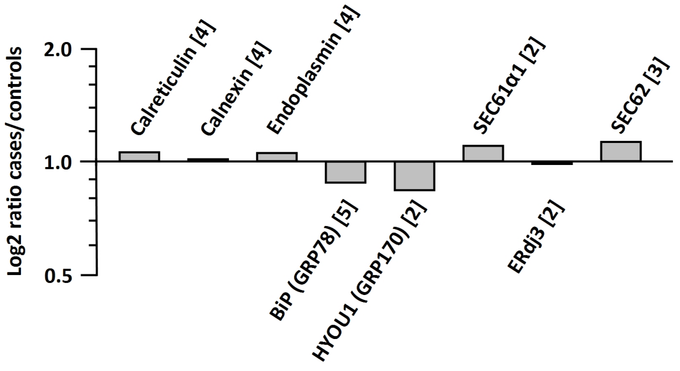

Skin fibroblasts from three individuals homozygous for the *INPP5K* mutation p.Ile50Thr (II.1, family C, III.1, family D and III.6, family D) and three healthy subjects were obtained from the MRC Centre for Neuromuscular Diseases Biobank, Newcastle, UK. Cells were cultured in DMEM containing 10% fetal calf serum, 2 mM glutamine, 40 U/mL penicillin and 0.04 mg/mL streptomycin. Cells were lysed in 1% SDS, 150 mM NaCl, 50 mM Tris-HCl, pH 7.8 containing protease and phosphatase inhibitors, and protein extracts were treated with benzonase (Merck Millipore) for 30 min at 37°C. Protein preparations were incubated at 95°C for 5 min, debris was removed by 30 min centrifugation at 18,000g at 4°C, and protein concentrations in the supernatants were measured with a BCA protein assay. Protein extracts were treated with 10 mM dithiothreitol for 30 min at 56°C followed by incubation with 30 mM iodoacetamide for 20 min at RT. Samples were digested with trypsin using filter-aided sample preparation [3-4] and then analyzed on an Ultimate 3000 nano RSLC system coupled to a Q Exactive HF mass spectrometer (Thermo Fisher Scientific) in parallel reaction monitoring mode. Peptides were pre-concentrated on a PepMap 100 µm x 2 cm C18 trapping column (Thermo Fisher Scientific) for 10 min using 0.1% trifluoroacetic acid at a flow rate of 20 µL/min, followed by separation on a PepMap 75 µm x 50 cm C18 main column (Thermo Fisher Scientific) with a 55 min gradient ranging from 5-42% of 84% acetonitrile, 0.1% formic acid at a flow rate of 250 nL/min. Mass spectra were acquired in the Orbitrap at a resolution of 30,000 with an automatic gain control target of 3 x 106, a maximum injection time of 100 ms, an isolation width of 0.4 m/z and a normalized collision energy of 27 in scheduled mode. Raw data was evaluated with Skyline software [5] and peak selection and assignment were manually validated. Unique peptides of proteins whose levels are altered under conditions of ER stress [6-7], calreticulin, calnexin, endoplasmin, BiP (GRP78), HYOU1 (GRP170), ERdj3, SEC61α1 and SEC62, were compared against an in-house spectral library. Peptide peak areas were used to determine median abundance of each protein of interest across all target protein-related peptides detected in two technical replicates of cases and controls. Median values were normalized to the median value calculated for proteome-wide peptides from the case or control group. Data are presented as the ratio cases median protein level/controls median protein level, plotted in logarithmic scale base 2. The counts of unique peptides for individual proteins are given in box brackets after the protein names.

Figure S10. Coprecipitation of BiP with INPP5K from transfected cells.

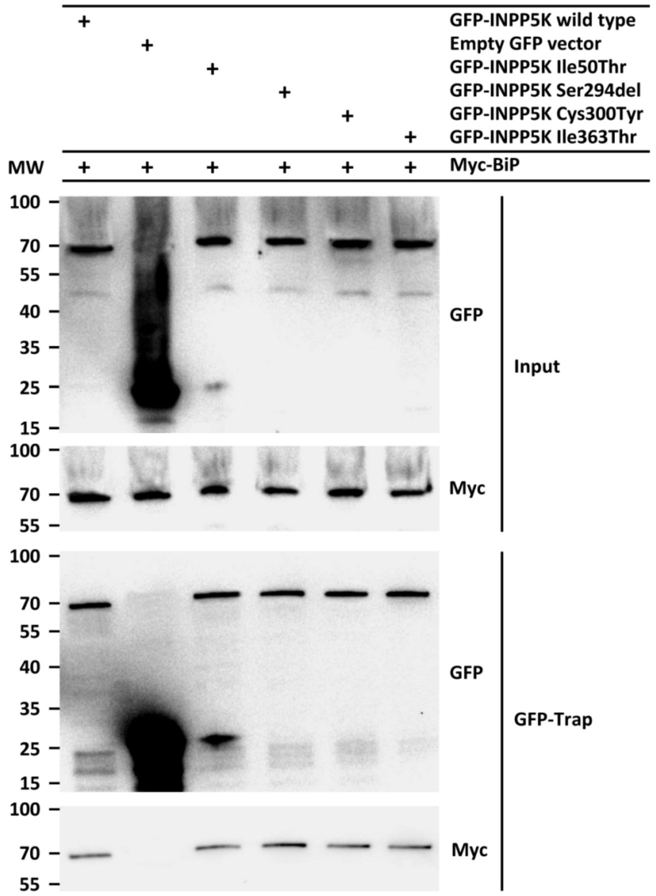

COS-7 cells were cotransfected with expression vectors for GFP-tagged INPP5K (or empty GFP vector) and Myc-tagged BiP (Addgene plasmid #27164, a gift from Ron Prywes [8]) using JetPei Transfection Reagent. Cells were lysed in 150 mM NaCl, 0.5 mM EDTA, 10 mM Tris-HCl, pH 7.5, 0.5% NP-40 supplemented with protease and phosphatase inhibitors. Protein extracts were incubated at 95°C for 5 min, debris was removed by 5 min centrifugation at 14,000 rpm at 4°C, and protein concentrations in the supernatants were measured with a BCA protein assay. Five hundred µg of total protein were mixed with GFP binding protein coupled to magnetic agarose beads (GFP-Trap [9]; ChromoTek) and left on a rotator for 1 hr at 4°C. Beads were magnetically separated, washed, resuspended in SDS sample buffer and boiled to elute bound proteins. Proteins were resolved by SDS-polyacrylamide gel electrophoresis, blotted onto PVDF membranes (Hybond; GE Healthcare) and transferred proteins were blocked for 1 hr at RT in TBS-T with 5% non-fat milk. To determine protein levels in transfected cells (Input) as well as amounts of precipitated GFP-INPP5K (or GFP alone) and bound Myc-BiP (GFP-Trap), membranes were incubated overnight at 4°C with primary antibodies mouse anti-Myc and rabbit anti-GFP followed by HRP-conjugated goat anti-mouse IgG and goat anti-rabbit IgG antibodies for 1 hr at RT. Bands were visualized with an ECL Detection Kit.

Figure S11. Measurement of autophagy in skeletal muscle and cultured skin fibroblasts.

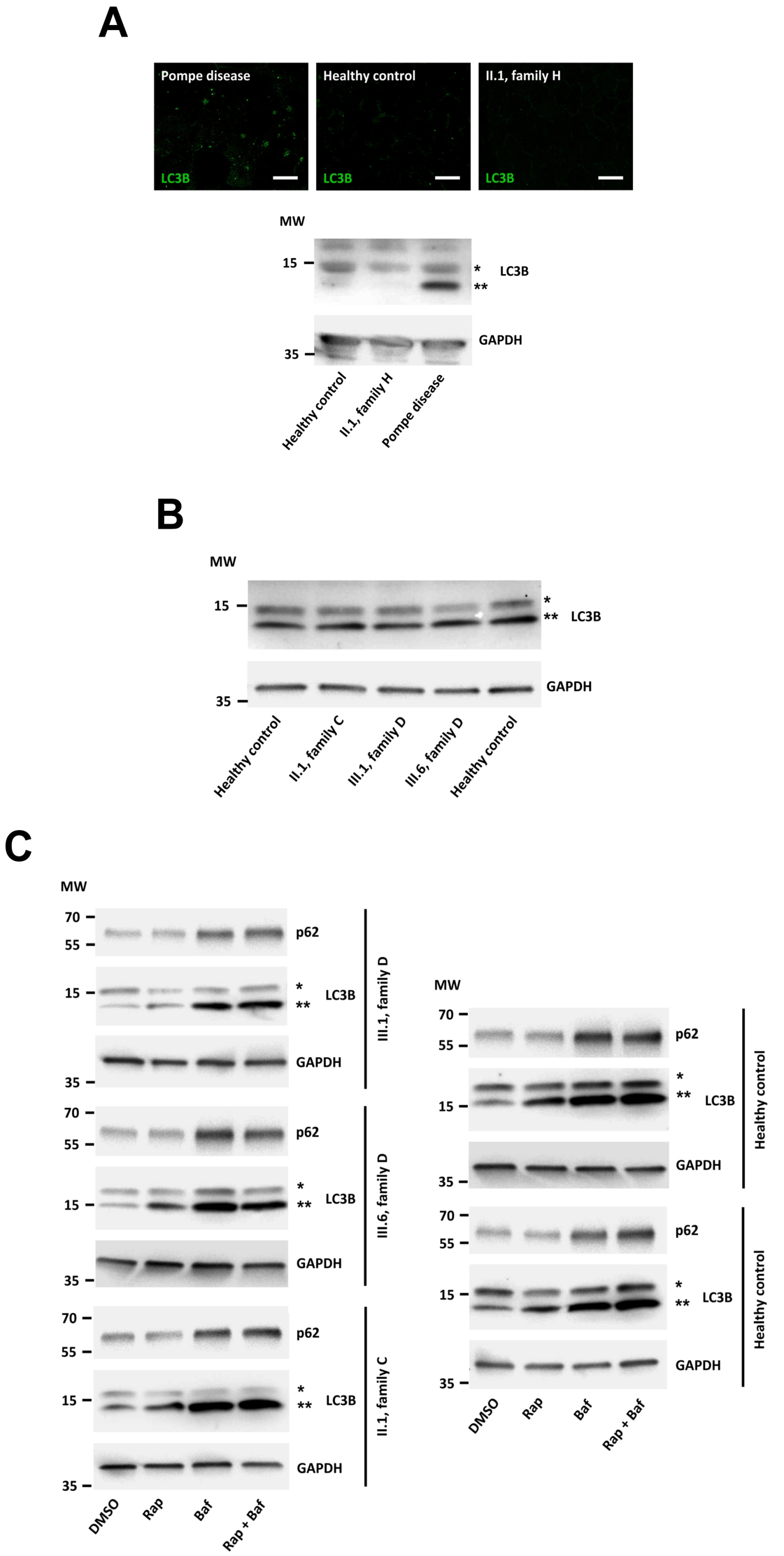

(A) Detection of LC3B in muscle biopsies from individual II.1, family H (homozygous *INPP5K* mutation p.Tyr300Cys), and healthy and pathological controls. Eight-µm cryostat sections of deep-frozen unfixed tissue samples were fixed with 4% paraformaldehyde, pH 7.4, for 10 min at RT, permeabilized with 0.1% Triton X-100 for 15 min and blocked in 5% horse serum in PBS for 1 hr at RT. Specimens were incubated at 4°C overnight with rabbit anti-LC3B antibody (Novus Biologicals; 1:100), followed (*continued on next page*)

(*legend to Figure S11, continued from previous page*) by Alexa Fluor 488-conjugated donkey anti-rabbit IgG antibody (Jackson ImmunoResearch; 1:500) for 1 hr at RT. Images were captured with a Zeiss Axiovert 200 M fluorescence microscope and a Zeiss AxioCam HR camera (Zeiss). For immunoblotting, skeletal muscle biopsy specimens were homogenized with a rotor-stator homogenizer in 1x PBS containing 1% SDS. Protein preparations were incubated at 95°C for 5 min, debris was removed by 5 min centrifugation at 14,000 rpm at 4°C, and protein concentrations in the supernatants were measured with a BCA protein assay. Equal amounts of total protein were separated on SDS-polyacrylamide gels and blotted onto PVDF membranes. Membranes were blocked for 1 hr at RT in TBS-T with 5% non-fat milk and then incubated overnight at 4°C with rabbit anti-LC3B (Novus Biologicals; 1:1,000) and rabbit anti-GAPDH antibodies. Subsequently, blots were incubated with HRP-conjugated goat anti-rabbit IgG antibody for 1 hr at RT. Bands were detected with an ECL Detection Kit. .* = LC3B-I, ** = LC3B-II. MW = Molecular weight in kilo Dalton. **(B)** Detection of LC3B in primary skin fibroblasts. Skin fibroblasts from three individuals homozygous for the *INPP5K* mutation p.Ile50Thr (II.1, family C, III.1, family D and III.6, family D) and two healthy subjects were obtained from the MRC Centre for Neuromuscular Diseases Biobank, Newcastle, UK. Cells were cultured in DMEM containing 10% fetal calf serum, 2 mM glutamine, 40 U/mL penicillin and 0.04 mg/mL streptomycin. Cells were lysed in radioimmunoprecipitation assay buffer (1% Triton X-100, 0.5% sodium deoxycholate, 1% SDS, 150 mM NaCl, 50 mM Tris-HCl, pH 7.4) containing protease and phosphatase inhibitors. Protein preparations were incubated at 95°C for 5 min, debris was removed by 5 min centrifugation at 14,000 rpm at 4°C, and protein concentrations in the supernatants were measured with a BCA protein assay. Equal amounts of total protein were run on SDS-polyacrylamide gels and transferred to PVDF membranes. Membranes were blocked for 1 hr at RT in TBS-T with 5% non-fat milk and then incubated overnight at 4°C with primary antibodies rabbit anti-LC3B and rabbit anti-GAPDH. Subsequently, blots were incubated with HRP-conjugated goat anti-rabbit IgG antibody for 1 hr at RT. Bands were detected with an ECL Detection Kit. MW = Molecular weight in kilo Dalton. **(C)** LC3B conversion and p62 levels in primary skin fibroblasts treated with autophagy modifiers. In addition to the procedures described in (B), cells were treated with 100 nM bafilomycin A1, 100 nM rapamycin or vehicle (DMSO) for 12 hr prior to cell lysis [10]. For detection of p62, a rabbit anti-p62 antibody (Sigma-Aldrich; 1:1,000) was used.

Table S1. Bioinformatic results for INPP5K amino acid substitutions.

Mutation	Mutation Taster prediction / score	PolyPhen-2 prediction / score	SIFT prediction / score	SNAP2 prediction / score	LRT prediction / LRT_{new} score	GERP++ score	PhyloP score
p.Ile50Thr	Disease causing / 1	Probably damaging / 0.999	Damaging / 0	Effect / 7	Deleterious / 1	5.4	1.062
p.Tyr300Cys	Disease causing / 1	Probably damaging / 1	Damaging / 0	Effect / 89	Deleterious / 1	5.53	0.991
p.Ile363Thr	Disease causing / 1	Probably damaging / 1	Damaging / 0	Effect / 22	Deleterious / 1	5.94	1.003

The score given by MutationTaster [11] is the probability of the prediction, i.e. a value close to 1 indicates a high "security" of the prediction. For PolyPhen-2 [12], scores near 1.0 are most strongly predicting a deleterious effect of an amino substitution. SIFT [13] scores ≤ 0.05 are assigned the prediction "damaging". SNAP2 [14] scores range from -100 strong neutral prediction to +100 strong effect prediction. Values for the LRT_{new} [15] score range from 0 to 1 with higher values indicating a variant is more likely to be deleterious. GERP++ [16] estimates evolutionary constraint of specific positions. Scores range from -12.3 to 6.17 with higher scores indicating more conserved sites. PhyloP [17] conservation scores are based on the multiple alignment of vertebrate genomes. Scores range from -5.172 to 1.062 and higher scores suggest stronger conservation of the site.

Supplementary References

1. Gudbjartsson, D.F., Jonasson, K., Frigge, M.L., and Kong, A. (2000). Allegro, a new computer program for multipoint linkage analysis. Nat Genet 25, 12-13.
2. Hartmann, W., Koch, A., Brune, H., Waha, A., Schüller, U., Dani, I., Denkhaus, D., Langmann., W., Bode, U., Wiestler, O.D., et al. (2005). Insulin-like growth factor II is involved in the proliferation control of medulloblastoma and its cerebellar precursor cells. Am J Pathol 166, 1153-1162.
3. Manza, L.L., Stamer, S.L., Ham, A.J., Codreanu, S.G., and Liebler, D.C. (2005). Sample preparation and digestion for proteomic analyses using spin filters. Proteomics 5, 1742-1745.
4. Wisniewski, J.R., Zougman, A., Nagaraj, N., and Mann, M. (2009). Universal sample preparation method for proteome analysis. Nat Methods 6, 359-362.
5. MacLean, B., Tomazela, D.M., Shulman, N., Chambers, M., Finney, G.L., Frewen, B., Kern, R., Tabb, D.L., Liebler, D.C., and MacCoss, M.J. (2010). Skyline: an open source document editor for creating and analyzing targeted proteomics experiments. Bioinformatics 26, 966-968.
6. Lee, A.S. (2014). Glucose-regulated proteins in cancer: molecular mechanisms and therapeutic potential. Nat Rev Cancer 14, 263-276.
7. Schroder, M., and Kaufman, R.J. (2005). The mammalian unfolded protein response. Annu Rev Biochem 74, 739-789.
8. Shen, J., Chen, X., Hendershot, L., and Prywes, R. (2002). ER stress regulation of ATF6 localization by dissociation of BiP/GRP78 binding and unmasking of Golgi localization signals. Dev Cell 3, 99-111.
9. Rothbauer, U., Zolghadr, K., Muyldermans, S., Schepers, A., Cardoso, M.C., and Leonhardt, H. (2008). A versatile nanotrap for biochemical and functional studies with fluorescent fusion proteins. Mol Cell Proteomics 7, 282-289.
10. Kimura, S., Fujita, N., Noda, T., and Yoshimori, T. (2009). Monitoring autophagy in mammalian cultured cells through the dynamics of LC3. Methods Enzymol 452, 1-12.
11. Schwarz, J.M., Rodelsperger, C., Schuelke, M., and Seelow, D. (2010). MutationTaster evaluates disease-causing potential of sequence alterations. Nat Methods 7, 575-576.
12. Adzhubei, I., Jordan, D.M., and Sunyaev, S.R. (2013). Predicting functional effect of human missense mutations using PolyPhen-2. Curr Protoc Hum Genet Chapter 7, Unit7 20.
13. Kumar, P., Henikoff, S., and Ng, P.C. (2009). Predicting the effects of coding non-synonymous variants on protein function using the SIFT algorithm. Nat Protoc 4, 1073-1081.
14. Hecht, M., Bromberg, Y., and Rost, B. (2015). Better prediction of functional effects for sequence variants. BMC Genomics 16 Suppl 8, S1.
15. Chun, S., and Fay, J.C. (2009). Identification of deleterious mutations within three human genomes. Genome Res 19, 1553-1561.
16. Davydov, E.V., Goode, D.L., Sirota, M., Cooper, G.M., Sidow, A., and Batzoglou, S. (2010). Identifying a high fraction of the human genome to be under selective constraint using GERP++. PLoS Comput Biol 6, e1001025.
17. Pollard, K.S., Hubisz, M.J., Rosenbloom, K.R., and Siepel, A. (2010). Detection of nonneutral substitution rates on mammalian phylogenies. Genome Res 20, 110-121.
